# Oxidative/Nitrative Stress and Inflammation Drive Progression of Doxorubicin-Induced Renal Fibrosis in Rats as Revealed by Comparing a Normal and a Fibrosis-Resistant Rat Strain

**DOI:** 10.1371/journal.pone.0127090

**Published:** 2015-06-18

**Authors:** Csaba Imre Szalay, Katalin Erdélyi, Gábor Kökény, Enikő Lajtár, Mária Godó, Csaba Révész, Tamás Kaucsár, Norbert Kiss, Márta Sárközy, Tamás Csont, Tibor Krenács, Gábor Szénási, Pál Pacher, Péter Hamar

**Affiliations:** 1 Semmelweis University, Institute of Pathophysiology, Budapest, Hungary; 2 National Institute of Health (NIH/NIAAA/DICBR), Laboratory of Physiological Studies, Section on Oxidative Stress and Tissue Injury, Bethesda, Maryland, United States of America; 3 University of Szeged, Faculty of Medicine, Department of Biochemistry, Szeged, Hungary; 4 1^st^ Semmelweis University, Department of Pathology and Experimental Cancer Research; MTA-SE Tumor Progression Research Group, Budapest, Hungary; University Medical Center Utrecht, NETHERLANDS

## Abstract

Chronic renal fibrosis is the final common pathway of end stage renal disease caused by glomerular or tubular pathologies. Genetic background has a strong influence on the progression of chronic renal fibrosis. We recently found that Rowett black hooded rats were resistant to renal fibrosis. We aimed to investigate the role of sustained inflammation and oxidative/nitrative stress in renal fibrosis progression using this new model. Our previous data suggested the involvement of podocytes, thus we investigated renal fibrosis initiated by doxorubicin-induced (5 mg/kg) podocyte damage. Doxorubicin induced progressive glomerular sclerosis followed by increasing proteinuria and reduced bodyweight gain in fibrosis-sensitive, Charles Dawley rats during an 8-week long observation period. In comparison, the fibrosis-resistant, Rowett black hooded rats had longer survival, milder proteinuria and reduced tubular damage as assessed by neutrophil gelatinase-associated lipocalin (NGAL) excretion, reduced loss of the slit diaphragm protein, nephrin, less glomerulosclerosis, tubulointerstitial fibrosis and matrix deposition assessed by periodic acid–Schiff, Picro-Sirius-red staining and fibronectin immunostaining. Less fibrosis was associated with reduced profibrotic transforming growth factor-beta, (TGF-β1) connective tissue growth factor (CTGF), and collagen type I alpha 1 (COL-1a1) mRNA levels. Milder inflammation demonstrated by histology was confirmed by less monocyte chemotactic protein 1 (MCP-1) mRNA. As a consequence of less inflammation, less oxidative and nitrative stress was obvious by less neutrophil cytosolic factor 1 (p47^phox^) and NADPH oxidase-2 (p91^phox^) mRNA. Reduced oxidative enzyme expression was accompanied by less lipid peroxidation as demonstrated by 4-hydroxynonenal (HNE) and less protein nitrosylation demonstrated by nitrotyrosine (NT) immunohistochemistry and quantified by Western blot. Our results demonstrate that mediators of fibrosis, inflammation and oxidative/nitrative stress were suppressed in doxorubicin nephropathy in fibrosis-resistant Rowett black hooded rats underlying the importance of these pathomechanisms in the progression of renal fibrosis initiated by glomerular podocyte damage.

## Introduction

Chronic kidney disease (CKD) is a major healthcare problem with a prevalence of 7% in Europe [1], and over 10% in the US according to the Centers for Disease Control and Prevention [2]. The pathologic manifestation of CKD is renal fibrosis, which is the final common pathway of many kidney diseases, such as diabetic and hypertensive nephropathy, toxic, ischemic or autoimmune renal diseases [3,4].

The clinical presentation of CKD varies widely among patients with the same initial disease [5]. The severity of symptoms and the rate of CKD progression are influenced by age, gender [6,7] and numerous pieces of evidence support a role for genetic background in progression [8,9,10]. We have demonstrated previously that Rowett, black hooded (BH) rats were resistant to renal fibrosis induced by subtotal nephrectomy plus salt and protein loading [11]. Better understanding of such resistance can shed light on the pathomechanisms of fibrosis in general and renal fibrosis specifically.

The anthracycline derivative chemotherapeutic drug, Doxorubicin (Adriamycin, DXR) is widely used as a rodent model of proteinuric nephropathy leading to renal fibrosis [12]. Although it is generally accepted that an initial injury to podocytes induces proteinuria, the exact pathomechanism of the DXR-induced nephropathy is poorly understood [13]. The role of sustained inflammation and oxidative stress has been demonstrated in many experimental models of renal fibrosis, including the remnant kidney [11,14,15] and DXR nephropathy models [12,16,17,18,19]. The myocardial and renal side effects of DXR are mainly attributed to the generation of free oxygen radicals [20]. DXR exerts direct toxic damage to the glomerular structure leading to loss of nephrin [21] and consequent proteinuria [22]. Proteinuria per se, sustained inflammation and accompanying oxidative damage are major mechanisms of progressive renal fibrosis [12]. It has been reported that the DXR-induced oxidative damage in cells of the renal cortex paralleled renal fibrosis progression [23]. DXR administration to rats led to severe tubulointerstitial inflammation with marked infiltration by T and B lymphocytes and macrophages. The intensity of inflammation correlated with the DXR-induced renal damage, and modifying pro-inflammatory pathways affected the severity of renal damage in this model [24,25,26].

We hypothesized that milder inflammation and milder accompanying oxidative/nitrative stress may be responsible for the previously published resistance of BH rats to renal fibrosis. To investigate the role of oxidative/nitrative stress and inflammation in the BH rats’ protection from renal fibrosis, we compared CD and BH rats in DXR nephropathy model.

## Materials and Methods

### Ethics Statement

Humane endpoints were used to minimize suffering in survival studies. Animals were observed and weighed every morning after potentially lethal interventions including DXR administration. If clinical signs of distress were recognized the animals were euthanized by cervical dislocation performed by a trained personnel. Uremic signs or body weight loss > 40% of the initial body-weight was an indication for euthanasia. Clinical signs of uremia are described later. Sacrifice for organ removal was performed under ketamine + xylazin (CP-Ketamin 10%, CP-Xylazin 2%, CP-Pharma, Burgdorf, Germany) anesthesia. All procedures were performed in accordance with guidelines set by the National Institutes of Health and the Hungarian law on animal care and protection. The experimental protocol was reviewed and approved by the “Institutional Ethical Committee for Animal Care and Use” of Semmelweis University (registration number: XIV-I-001/2104-4/2012).

### Animals and experimental design

Eight-week-old male Charles Dawley (CD) and Rowett, black hooded (BH) rats were used in the studies (Charles River, Hungary). After arrival the animals were allowed 1 week for acclimatization. All animals were maintained under standardized (light on 08:00–20:00 h; 40–70% relative humidity, 22±1°C), specified pathogen-free (SPF) conditions, with free access to water and standard rodent chow (Altromin standard diet, Germany).

We performed the following three experiments:

1. Renal functional and morphological experiment in DXR-induced acute renal failure;
2. Long term survival study with low dose DXR;
3. Short term survival study with high dose DXR.

In the functional and morphological experiment (exp. 1) rats (n = 8/group) were intravenously injected with 5 mg/kg body weight DXR (Sicor S.r.l. Società Italiana Corticosteroidi, Italy) dissolved in saline. Equal volume of saline was administered to control animals. DXR dose was based on literature data and pilot experiments. In the pilot experiments 2 mg/kg DXR did not induce renal damage, whereas 8 mg/kg DXR caused premature moribund state in some animals. Urinary protein and NGAL excretion was followed for 8 weeks when the experiment was terminated and renal morphology was investigated. Long-term survival (exp. 2) was evaluated in age matched BH and CD rats (n = 8/group) (5 mg/kg DXR, iv). For short-term survival (exp. 3) 10 mg/kg DXR was injected iv (n = 8/group). In survival experiments animals were euthanized upon signs of uremia, which included reduced locomotion, pilo-erection, body weight loss or dyspnoea. Blood urea was > 250 mg/dl in each euthanized animal demonstrating that uremia was the cause of the moribund state.

In order to investigate whether the difference in the degree of tubulointerstitial fibrosis between the two rat strains was the consequence of different tubular protein load, or BH rats were resistant to tubulointerstitial fibrosis per se, we formed two sub-groups. In this analysis CD and BH rats treated with DXR (CD/DXRp, n = 4 and BH/DXRp, n = 5) were matched for urinary protein excretion and sensitive molecular, inflammatory and fibrosis parameters were compared.

### Proteinuria and NGAL excretion

Proteinuria was measured as a sensitive indicator of podocyte injury and progression of renal fibrosis. Urine was collected for 24 hours in diuresis cages (Techniplast, Italy) before (self-control) and biweekly after DXR administration. Urine protein concentration was assessed with a pyrogallol red colorimetric assay (Diagnosticum Ltd, Budapest, Hungary). Optical density was measured at 598 nm with the SpectraMax 340 Microplate Spectrophotometer (Molecular Devices, Sunnyvale, USA).

Urine NGAL levels were measured with rat Lipocalin-2/NGAL DuoSet ELISA Development kit (R&D Systems, USA) as described by the manufacturer. Optical density was measured with Victor3 1420 Multilabel Counter (PerkinElmer, WALLAC Oy, Finland) at 450 nm with wavelength correction set to 544 nm. Concentrations were calculated with WorkOut (Dazdaq Ltd., England), using a four parameter logistic curve-fit.

### Sacrifice and renal sample collection

In the functional and morphological study (exp. 1), rats were anesthetized with ketamine + xylazine 8 weeks after DXR administration. To prevent blood clotting, 1 ml/kg Na-EDTA (Sigma-Aldrich Corporation, Saint Louis, MO, USA) was injected intraperitoneally. Rats were bled from the aorto-femoral bifurcation. Animals were perfused through the aorta with 60 ml cold physiological saline to remove blood from the vasculature. After perfusion, the left kidney and the heart were removed and sectioned for further analysis. The heart and a third of the left kidney were fixed in 4% buffered formaldehyde and were later embedded in paraffin for basic histological and immunohistochemical analysis. The remaining two third of the left kidney cortex and medulla were separated, frozen in liquid nitrogen and stored at -80°C for molecular studies.

### Renal morphology

Glomerulosclerosis was assessed according to a modified [11,27] scoring system (scores 0–4) of El Nahas et al. [28] at x400 absolute magnification using an Olympus CX21 microscope (Olympus Optical Co. Ltd., Japan). Score 0: normal glomerulus. Score 1: thickening of the basal membrane. 2: mild (<25%), 2.5: severe segmental (>50%) and 3: diffuse hyalinosis. 4: total tuft obliteration and collapse. The glomerular score of each animal was derived as the arithmetic mean of 100 glomeruli.

Tubulointerstitial damage was assessed with a semi quantitative scale (magnification ×100) of percent area affected by tubulointerstitial changes [21,29]. Score 0: normal tubules and interstitium, 1: brush border loss or tubular dilatation in <25% of the field of view (fv). 2: tubular atrophy, dilation and casts in < 50% fv. Score 3: tubular and interstitial damage in < 75% fv, 4: tubular atrophy, dilation, casts and fibrosis > 75% fv. The overall score was the mean of 15 fvs.

Inflammatory infiltration was assessed on hematoxylin-eosin stained sections by the percent of area infiltrated by inflammatory cells (magnification: x400). Score 0: normal glomeruli, tubules and interstitium, 1: inflammatory cells present in <25% fv. 2: inflammation in < 50% fv. Score 3: inflammation in < 75% fv, 4: inflammation in > 75% of fv. The overall score was the mean of 120 fvs.

**Collagen deposition** in the renal interstitium was demonstrated by Picro-Sirius Red staining as described previously. Fibrotic areas were quantified using Image J software (National Institutes of Health, Bethesda, Maryland, US).

#### Antibodies

For Western blot and immunohistochemistry: 4-hydroxy-2-nonenal (HNE, mouse monoclonal, clone: HNEJ-2, JaICA, Japan), NT (mouse monoclonal, #189542, Cayman Chemical Company, Michigan, IL), fibronectin (rabbit polyclonal, Sigma-Aldrich, Budapest, Hungary), Connexin-43 (1:100, #3512, Cell Signaling, Beverly, MA) were used.

#### Western Blot

The kidney samples were lysed in RIPA Buffer (Thermo Scientific, Rockford, IL). Protein concentration was determined by the bicinchoninic acid (BCA) protein assay (Thermo Scientific, Rockford, IL). Twenty μg protein was resolved on 4–12% Criterion XT Bis-Tris Precast gels (BioRad, Hercules, CA) and transferred to nitrocellulose membrane to detect HNE or to Polyvinylidene Difluoride (PVDF) membrane to detect NT. The primary NT antibody was applied at 1.3 μg/mL and the primary HNE antibody at 0.3 μg/mL. The secondary antibody (peroxidase conjugated goat anti-mouse, PerkinElmer, Santa Clara, CA) was applied at 0.25 μg/mL. Blots were incubated in enhanced chemiluminescence substrate, Supersignal West Pico Chemiluminescent Substrate (Thermo Scientific, Rockford, IL), and were exposed to photographic film. After stripping membrane with Restore Western Blot Stripping Buffer (Thermo Scientific, Rockford, IL), as a loading control, peroxidase conjugated anti-actin (AC-15 Abcam, Cambridge, MA) was applied at 70 ng/mL concentration in blocking buffer for 1 h at room temperature.

#### Immunohistochemistry

Paraffin sections on Superfrost Ultra Plus Adhesion Slides (Thermo Fisher Scientific Inc, Waltham, MA, USA) were deparaffinized and rehydrated in ethanol. Fibronectin immunohistochemistry was performed with rabbit polyclonal anti-fibronectin antibody (1:2000, Sigma-Aldrich, Budapest, Hungary), using the avidin–biotin method [30]. HNE and NT immunohistochemistry was performed with mouse monoclonal antibody (HNE clone: HNEJ-2, JaICA, Japan; NT clone: #189542, Cayman Chemical Company, Michigan, IL). Color development was induced by incubation with diaminobenzidine (DAB) kit (Vector Laboratories, Burlingame, CA). Pictures were taken from the stained sections for further analysis. The fibronectin stained area was quantified with Image J software.

### Heart fibrosis markers

In a separate group, the hearts were removed and fixed in 4% buffered formalin and embedded similarly to the renal samples 8 weeks after 5 mg/kg DXR administration. Consecutive sections were stained with Masson’s trichrome to detect collagen deposition as a sign of chronic fibrosis, and direct immunofluorescence was performed for connexin-43 (Cx43), an early marker of cardiomyocyte damage.

### Monitoring mRNA levels with Real-Time quantitative Polymerase Chain Reaction (RT-qPCR)

#### RNA preparation

Total RNA for RT-qPCR was extracted by homogenizing 50–80 mg pieces of renal cortex in TRI Reagent (Molecular Research Center Inc., Cat. NO.: TR118) according to the manufacturer’s protocol. DNA contamination was removed by TURBO DNase (Life technologies, Ambion, Cat. No: AM2238). RNA concentration and purity of the samples was measured with the NanoDrop 2000c Spectrophotometer (Thermo Fisher Scientific Inc, Waltham, MA, USA). The RNA integrity was verified by electrophoretic separation on 1% agarose gel.

#### RT-qPCR analysis of renal mRNA levels

Reverse transcription of 1 μg total renal RNA into cDNA was carried out using random hexamer primers and the High-Capacity cDNA Archive Kit (Applied Biosystem, USA) according to the manufacturer’s protocol. Messenger RNA levels of NADPH oxidase-2 (NOX-2, p91^phox^, cytochrome b-245 beta polypeptide), neutrophil cytosolic factor 1 (Ncf1, p47^phox^), collagen type I, alpha 1 (COL1A1), transforming growth factor β1 (TGF-β1), connective tissue growth factor (CTGF) and macrophage chemotactic protein 1, (MCP-1, chemokine (C-C motif) ligand 2, Ccl2) were measured by RT-qPCR (Qiagen, Hilden, Germany) and target mRNA levels were normalized to actin mRNA levels (Table 1).

**Table 1:** Qiagen primer reference numbers.

Gene	Reference sequence	Qiagen primer reference number
p91^phox^ (NOX2)	NM_023965.1	QT00195300
p47^phox^ (Ncf1)	NM_053734	QT00189728
MCP-1 (Ccl2)	NM_031530.1	QT00183253
TGF-β1	NM_021578.2	QT00187796
CTGF	NM_022266.2	QT00182021
COL1A1	NM_053304.1	QT02285619

Nephrin mRNA levels were measured by double-stranded DNA (dsDNA) dye based RT-qPCR with Maxima SYBR Green RT-qPCR Master Mix (Thermo Fisher Scientific Inc., Waltham, MA, USA), and the mRNA values were normalized to glyceraldehyde-3-phosphate dehydrogenase. Mean values are expressed as fold mRNA levels relative to the control using the formula *2* ^*-*Δ(Δ*Ct*)^ (Ct: cycle time, ΔCT = CT_target_−CT_normalizer_ and Δ(ΔCT) = ΔCT _stimulated_- ΔCT_control_) [31].

### Statistics

Two-way ANOVA with or without repeated measures were used for multiple comparisons. Post hoc analyses were done with Holm-Sidak‘s test. Logarithmic transformation of data was used if Bartlett’s test indicated a significant inhomogeneity of variances. Variables of the two sub-groups within the BH/DXR and the CD/DXR groups were compared using unpaired t-test. Survival was analyzed according to the Kaplan-Meier method. The null hypothesis was rejected if p < 0.05. Data were expressed as means ±SEM if not specified otherwise. All statistical analysis was done with GraphPad Prism (version 6.01, GraphPad Software Inc, San Diego, CA, USA).

## Results

### Heart toxicity was absent 8 weeks after 5 mg/kg DXR

Histology of the heart did not show necrosis or other morphological alterations of cardiomyocytes. Massons’s trichrom staining was devoid of collagen deposition, and connexin-43 immunostaining did not demonstrate any sign of cardiomyocyte damage. Thus, a single dose of 5 mg/kg DXR did not induce any detectable chronic heart damage (data not shown).

### CD rats became moribund earlier than BH rats at both low and high doses of DXR

**Fig 1:**
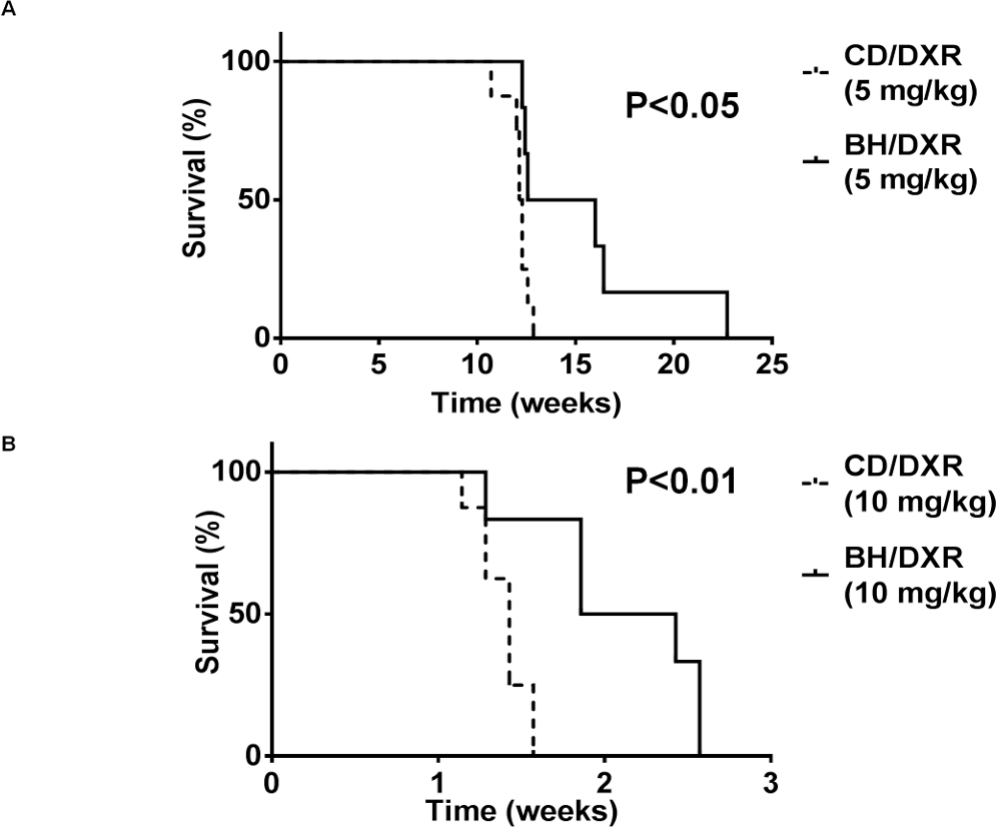
Survival (long term: A, short term: B). CD: CD rats, BH: BH rats. DXR: Doxorubicin injected rats (5 mg/kg).

BH rats became moribund significantly later following the 5 mg/kg DXR dose, compared to CD rats (Fig 1A). The first CD rat became moribund 75 days after DXR administration, and there were no survivors after day 90 from this strain. The first BH became moribund 86 days after DXR, and there were survivors even 159 days after DXR administration. The median survival after DXR was 85.5 days for the CD rats, while it was 100 days for the BH rats (p<0.05).

A higher dose of 10 mg/kg DXR led to a more severe outcome. The median survival of CD rats was 10 days compared to 15 days of the BH rats (Fig 1B). DXR administration caused less severe kidney damage than subtotal nephrectomy (SNX) and salt + protein loading in our previous study [11] as demonstrated by longer survival.

### DXR inhibited bodyweight gain more in CD than in BH rats

**Fig 2:**
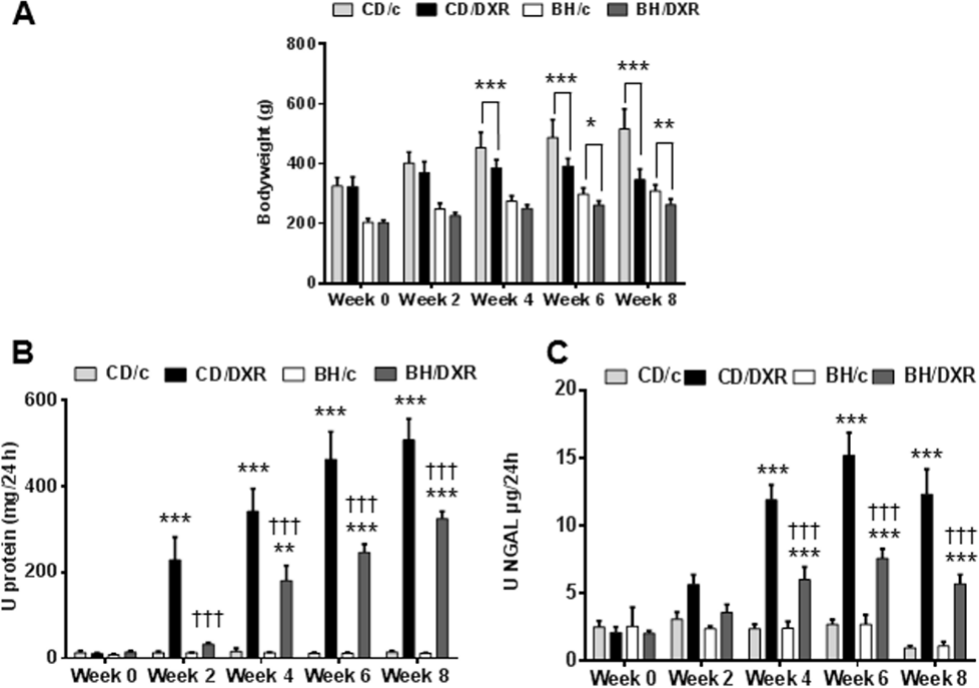
Body weight changes (A) proteinuria (B) and urinary NGAL excretion (C). CD: CD rats, BH: BH rats. c: saline-injected, DXR: Doxorubicin-injected rats (5 mg/kg). *: p<0.05 vs. strain-identical, negative control group, †: p<0.05 vs. CD/DXR, positive control group.

DXR-administration inhibited weight gain in BH and CD rats (Fig 2A). BH rats had a slower growth rate than age matched CD rats. Bodyweight constantly increased in all control animals. Body weight gain was significantly inhibited in DXR-injected CD rats (CD/DXR) already starting at week 4 while CD/DXR rats started to lose weight by week 8. On the contrary, significant weight gain inhibition was observed in BH rats (BH/DXR) only at week 8.

### Proteinuria was milder in BH than CD rats after DXR

Proteinuria was assessed as a marker of podocyte damage and progression of renal fibrosis. In the functional and morphological experiment (exp. 1) 5 mg/kg DXR induced progressive proteinuria commencing 2 weeks after DXR in CD rats (Fig 2B). Proteinuria started later and progressed slower in BH than in CD rats, and proteinuria was significantly milder at each time-point in BH/DXR than in CD/DXR rats.

Urinary NGAL excretion—a known marker of tubular epithelial damage—increased in both DXR-injected groups after the fourth week. Similarly to proteinuria, NGAL excretion was significantly milder in the BH/DXR than in the CD/DXR group (Fig 2C).

### Renal histological damage and inflammation were more severe in CD than in BH rats 8 weeks after DXR

**Fig 3:**
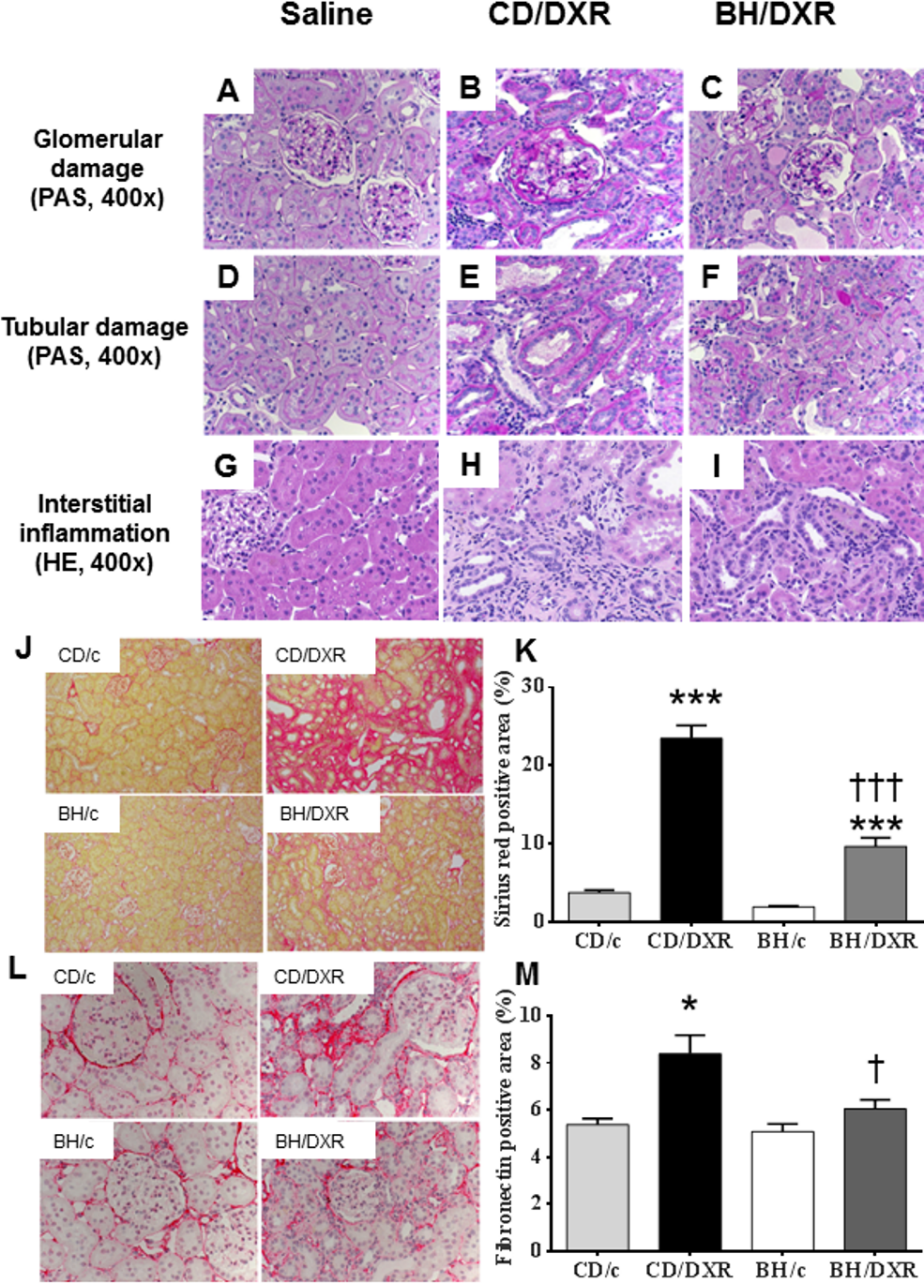
Renal histopathology. Top row (periodic acid–Schiff (PAS) staining) (**A-C**): Glomerular damage in the affected areas. Middle row PAS staining) (**D-F**): Tubular damage in the affected areas. Lower row (hematoxylin-eosin (HE) staining) (**G-I**): Tubulointerstitial inflammatory infiltration. Saline-injected control rats (**A,D,G**); CD-DXR: doxorubicin-injected (5 mg/kg) CD rats (**B,E,H**); BH-DXR: doxorubicin-injected BH rats (**C,F,I**). **J:** Sirius red staining (100x). **L:** Fibronectin immunohistochemistry (400x). **K, M:** computerized quantification of the immunostained areas. CD: CD rats, BH: BH rats. c: saline-injected, DXR: Doxorubicin-injected rats (5 mg/kg). *: p<0.05 vs. strain-identical, control group, †: p<0.05 vs. CD/DXR, control group.

Both CD and BH rats injected with saline had normal kidneys with no or minimal glomerular and tubular abnormalities 8 weeks after the injection. DXR administration caused glomerular damage in all age-matched rats. However, BH/DXR rats had milder glomerular and tubular damage compared to CD/DXR rats (Fig 3A–3F, Table 2). Glomerular and tubular damage were distributed unevenly in rats, similarly to human focal segmental glomerulosclerosis (FSGS) [18]. In parallel with milder proteinuria and urinary excretion of NGAL in BH rats, intact glomeruli (Score: 0) were significantly more common in DXR-injected BH than in CD rats (Table 2). However, mild (Score: 0.5–1.5) (CD: 50.7 vs. BH: 28.4%) and severe (Score ≥ 2) (CD: 13.3 vs. BH: 3.3%) glomerular damage was significantly more common in CD/DXR vs. BH/DXR rats. Probably as a consequence of different degrees of glomerular damage, tubulointerstitial damage was milder in BH than in CD rats (Table 2).

**Table 2:** Renal morphology.

Groups	Undamaged glomeruli (%)	Glomerulosclerosisscore	Tubular score	Inflammation score
**CD/DXR**	36.3±13.4	0.79±0.22	2.01±0.64	1.61±0.32
**BH/DXR**	68.3±8.4	0.32±0.11	0.86±0.44	1.06±0.20
**Control**	93.3±4.4	0.06±0.04	0.00±0.00	0.18±0.06
**P value**(CD/DXR vs. BH/DXR)	<0.001	<0.001	<0.001	<0.01

CD: CD rats, BH: BH rats. Control: saline-injected, DXR: Doxorubicin-injected rats (5 mg/kg). Mean±SD, n = 10/group.

Eight weeks after DXR administration severe inflammatory infiltration by neutrophil granulocytes, lymphocytes and macrophages was evident in the kidney samples of DXR-injected CD rats. In parallel with less proteinuria and morphological damage, inflammation was significantly milder in BH/DXR rats than in CD rats (Fig 3G–3I).

### Milder fibrosis was associated with less oxidative stress and inflammation

**Fibrosis** was strikingly more intense in CD/DXR than in BH/DXR rats as demonstrated by Sirius red staining (Fig 3J and 3K). Fibronectin immunostaining was detected only in 5.2±0.6% of the scanned areas in the saline-injected control groups, but increased significantly in the DXR-injected CD group. Significantly less fibronectin staining was detected in BH/DXR than in CD/DXR rats (Fig 3L and 3M).

**Fig 4:**
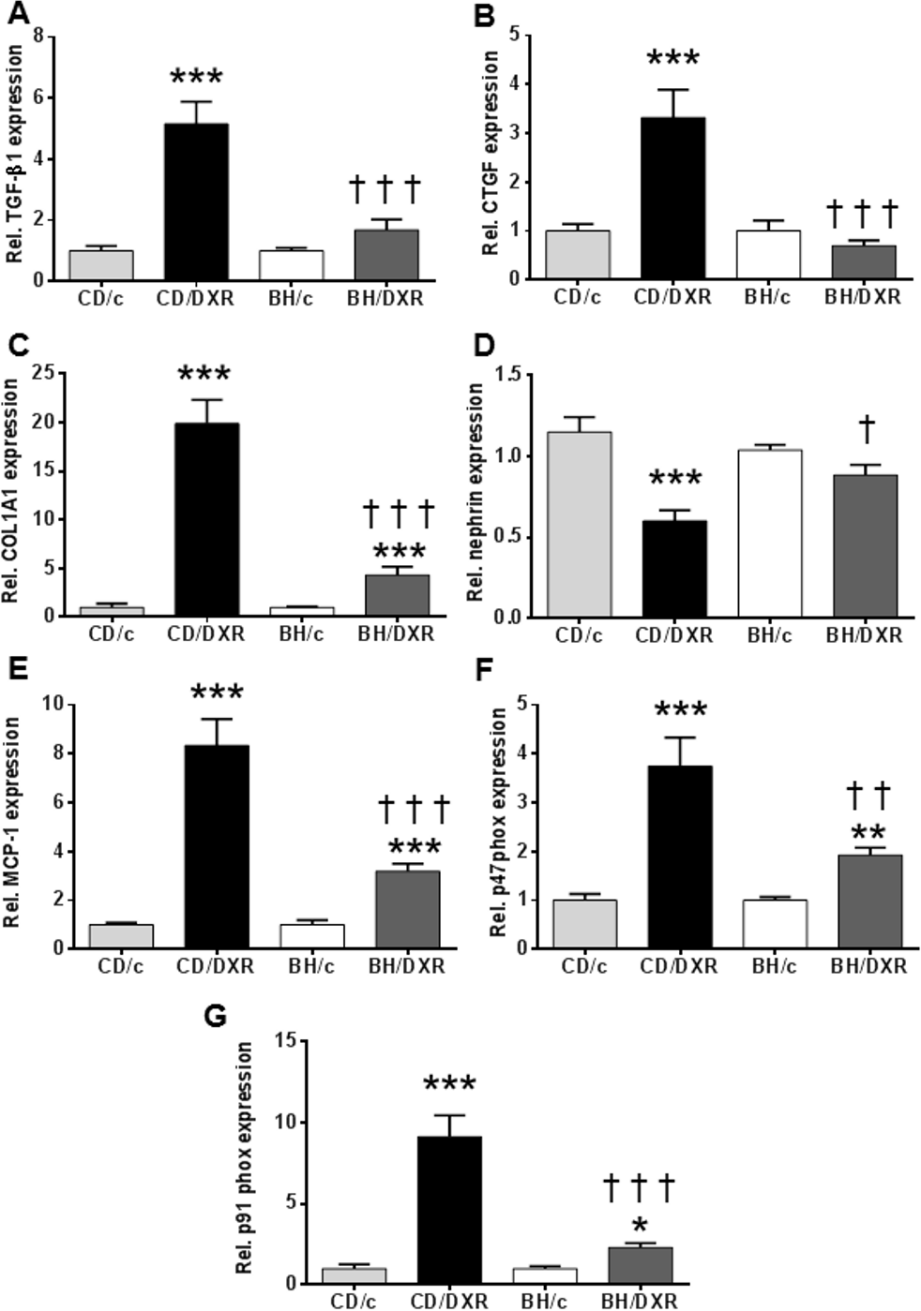
Renal cortical mRNA levels of fibrosis related, inflammatory and oxidative markers. (**A:** TGF-β1, **B:** CTGF, **C:** COL1A1, **D:** nephrin, **E:** MCP-1 **F:** p47^phox^, **G**: p91^phox^. CD: CD rats, BH: BH rats. c: saline-injected, DXR: Doxorubicin-injected rats (5 mg/kg). TGF-β1: transforming growth factor β1; CTGF: connective tissue growth factor; COL1A1: collagen type I alpha 1; MCP-1: monocyte chemotactic protein 1; p47^phox^: neutrophil cytosolic factor 1; p91^phox^: cytochrome b-245, beta polypeptide; *: p<0.05 vs. strain-identical control group, †: p<0.05 vs. CD/DXR.

**TGF-β1 and CTGF mRNA levels** in the kidney cortex were not significantly different in the control groups compared to DXR-injected BH rats, but were significantly elevated in the CD/DXR group (Fig 4A and 4B). COL1A1 mRNA levels were elevated in DXR-injected rats, but the elevation was significantly higher in the CD/DXR group than in the BH/DXR group (Fig 4C).

**Nephrin** is an important component of the podocyte foot processes forming the slit diaphragm. It plays an important role in the maintenance of the structural integrity and the functional soundness of the slit diaphragm [32]. Nephrin mRNA levels decreased in the kidney cortex of the CD/DXR group, but it was not reduced in the BH/DXR group (Fig 4D) supporting further a milder glomerular damage in BH/DXR rats.

**The mRNA levels of pro-inflammatory monocyte chemotactic protein 1** (MCP-1) (Fig 4E) and pro-oxidant markers: p91^phox^ and p47^phox^ (Fig 4F and 4G) increased in both DXR-injected groups; however the elevation was milder in the BH/DXR group.

#### 4-hydroxy-2-nonenal and nitrotyrosine

**Fig 5:**
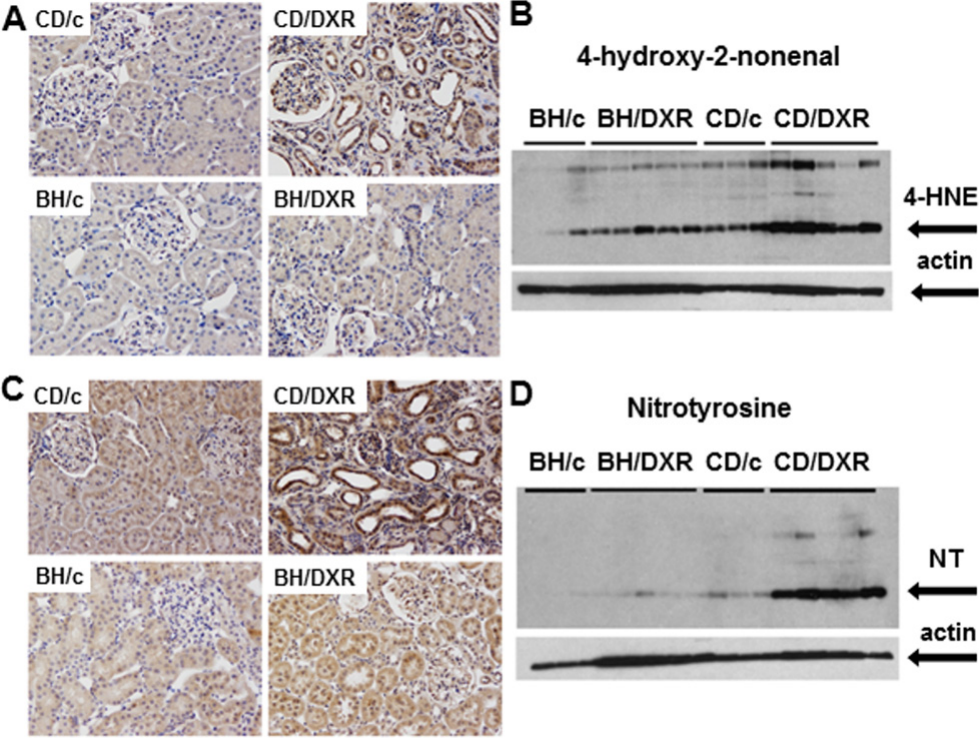
Oxidative/nitrative stress markers. **A:** 4-hydroxy-2-nonenal (HNE) immunohistology (400x) and **B**: quantification by Western blot. **C:** Nitrotyrosine (NT) immunohistology (400x) and **D:** quantification by Western blot. CD: CD rats, BH: BH rats. c: saline-injected, DXR: Doxorubicin-injected rats (5 mg/kg).

In the background of more severe kidney function deterioration demonstrated by proteinuria and fibrosis markers, severe lipid peroxidation and nitrative stress were detected in the kidneys of DXR-injected CD rats, while very mild changes were seen in the HNE or NT stained paraffin sections from the BH/DXR rats. Less staining was corroborated by Western blot demonstrating significantly more HNE and NT in CD than in BH rats 8 weeks after DXR administration (Fig 5).

### Tubulointerstitial fibrosis and inflammation were milder in DXR-treated BH vs. CD rats despite similar proteinuria

Urinary protein excretion and renal nephrin mRNA levels were similar in the two subgroups of CD and BH rats (BH/DXRp, CD/DXRp) with similar proteinuria (Table 3). Markers of fibrosis such as sirius red staining and relative renal expression of TGF-β1, CTGF, COL1A1 were significantly lower in BH/DXRp vs. CD/DXRp rats. Paralleling less fibrosis, tubular damage detected by urinary NGAL excretion, markers of oxidative damage such as p47^phox^ and p91^phox^ expression and inflammation (MCP-1 expression) were significantly lower in BH/DXRp than CD/DXRp rats (Table 3). All raw data are available in S1 Data Supplement.

**Table 3:** Comparison of doxorubicin-injected (DXR) Rowett, black hooded (BH) and Charles Dawley (CD) rats with similar proteinuria (BH/DXRp and CD/DXRp subgroups).

	CD/DXRp (n = 4)	BH/DXRp (n = 5)	P value
**U Protein, week 8 (mg/24h)**	396.5±82.2	362.5±30.3	0.42
**Nephrin**	0.68±0.16	0.87±0.19	0.19
**U NGAL, week 8 (mg/24h)**	10.1±2.0	5.2±1.6	**<0.01**
**Sirius red (%)**	18.0±1.6	11.8±1.0	**<0.01**
**Fibronectin (%)**	7.91±2.45	5.55±1.19	0.16
**TGF-β1**	6.00±2.36	2.13±1.23	**<0.05**
**CTGF**	3.71±2.10	0.54±0.11	**<0.05**
**COL1A1**	23.09±6.14	5.79±2.41	**<0.01**
**p47** ^**phox**^	4.69±1.51	1.95±0.36	**<0.05**
**p91** ^**phox**^	10.69±2.47	1.94±0.78	**<0.01**
**MCP-1**	9.23 3.28	3.46±0.99	**<0.05**

## Discussion

Renal fibrosis is an intractable medical condition with high mortality and low quality of life. We present here an animal model useful to investigate the pathomechanisms of hereditary susceptibility or resistance to renal fibrosis in various kidney injury models [10,33,34]. We demonstrated recently that BH rats were resistant to renal fibrosis with better preserved renal function and glomerular structure in a clinically relevant model of subtotal nephrectomy combined with salt and protein loading [11]. In the present study we demonstrate that less oxidative/nitrative stress and inflammation was associated with slower progression of fibrosis in the resistant strain. Taken together with our previous report demonstrating similar resistance of BH vs. CD rats in the subtotal nephrectomy model, our present findings underline the pathophysiological relevance of inflammation and oxidative/nitrative stress pathways in fibrosis progression.

DXR nephropathy in rodents is a widely used experimental model of human FSGS [12,35]. Direct exposure of the kidneys to DXR is a requirement for the development of podocyte injury in rats, as clipping the renal artery during DXR injection prevents nephropathy [35]. A single intravenous injection with 4–7,5 mg/kg DXR led to well predictable deterioration of glomerular structure, proteinuria, tubular and interstitial inflammation culminating in renal fibrosis in fibrosis-sensitive Sprague Dawley or Wistar rats [36]. Glomerular structural changes develop in a well predictable manner: altered mRNA levels of nephrin, podocyn and NEPH1, and swelling of the foot-processes are present at day 7 [37]. Podocyte swelling with cytoplasmic vesicles appear at day 14, and finally widespread podocyte foot process fusion at day 28 [38]. As a marker of glomerular filtration barrier damage, proteinuria develops [39]. Repeated low dose DXR has been widely used to induce toxic cardiomyopathy. In our model cardiac toxicity was absent after a single DXR injection, as demonstrated by histology or the sensitive cardiomyopathy marker Cx-43.

BH rats have a slower growth rate than age matched CD rats under healthy circumstances. The body weight curve in control animals of our study were similar to the previous findings [40,41].

**Fig 6:**
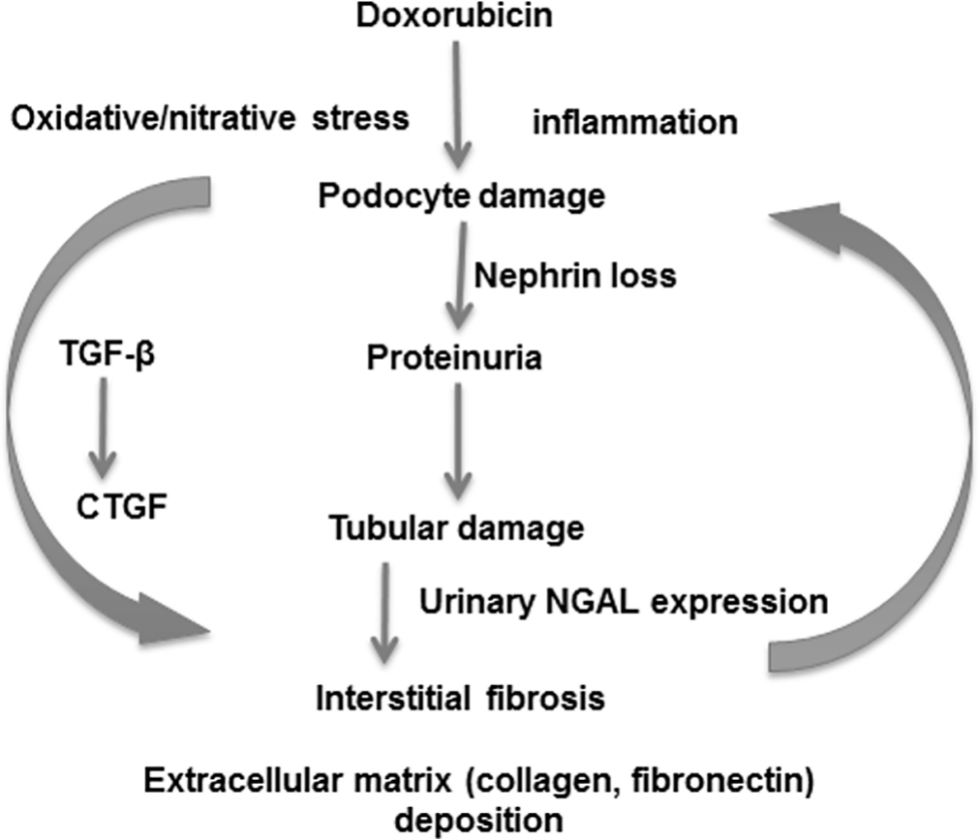
Suggested mechanisms of doxorubicin induced nephropathy. A single administration of doxorubicin induced podocyte damage demonstrated by loss of nephrin and leading to proteinuria. Proteinuria damages tubules as demonstrated by increased urinary NGAL excretion. Tubular damage leads to interstitial inflammation and fibrosis with collagen and fibronectin deposition. Inflammation is accompanied by oxidative/nitrative damage triggering further immune activation. Reverse arrows symbolize main elements of the vicious circle. Sustained injury activates the TGF-β1 and CTGF profibrotic axis. Sustained injury eventually leads to fibrotic end-stage kidney. NGAL: neutrophil gelatinase-associated lipocalin; TGF-β1: transforming growth factor β1; CTGF: connective tissue growth factor.

In our study, sensitive CD rats developed significant and progressive proteinuria starting two weeks after 5 mg/kg DXR similarly to that shown in previous publications [37–42]. Nephrin plays an important role in maintaining the structural integrity and the functional soundness of the slit diaphragm [32]. In the background of the proteinuria significant nephrin loss was demonstrated in CD rats. The severity of proteinuria was milder and progression was slower in BH rats. Thus, as BH rats had similar nephrin mRNA levels to the strain-identical controls, nephrin might play a central role in the progression of DXR-induced fibrosis. Proteinuria-associated interstitial fibrosis and tubular atrophy (IFTA) has been recognized previously [43]. Podocyte dysfunction and consequent proteinuria has been recently reinforced as a major determinant of tubular injury, inflammation and apoptosis leading to progressive IFTA [44]. According to our present study and previous literature [45] IFTA developed as part of DXR nephropathy. Urinary NGAL excretion is a sensitive marker of tubular damage not only during acute kidney injury [[46, 47],] but also during IFTA [48]. In our study, significantly less proteinuria was accompanied by reduced tubular damage and less urinary NGAL excretion after DXR in the BH than in the CD strain. Similarly, less renal damage and less proteinuria was accompanied by better maintained body weight and significantly prolonged survival in BH rats. These data support that IFTA is secondary to proteinuria in the DXR model. The single administration of DXR and consequent albuminuria led to tubulointerstitial inflammation and fibrosis demonstrated by PAS, Sirius red and fibronectin and collagen synthesis and the presence of the pro-fibrotic transforming growth factor (TGF-β1) [[49, 50],] and its downstream mediator connective tissue growth factor (CTGF) [51]. Significant reduction of these fibrotic pathways in the resistant BH strain underlines the relevance of the TGF-β1-CTGF cascade-mediated matrix deposition in the development of DXR-induced renal fibrosis (Fig 6).

Oxidative and nitrative stress has been proposed as the mechanism by which DXR induces glomerular toxicity in rats. Redox cycling of the quinone functional group of DXR was proposed as the key factor in DXR nephrotoxicity [52]. Reactive oxygen species (ROS) may initiate a degenerative cascade by the oxidation of cellular thiols and lipid membrane structures [53]. DXR has been suggested to upregulate NADPH-oxidase (NOX), an important source of ROS in the kidney [54]. However, the role of oxidative mechanisms in DXR toxicity has been questioned as well [55]. In our study, signs of lipid peroxidation and nitrative stress were milder in the BH rats, compared to the CD rats suggesting that less oxidative and nitrative stress may be responsible, at least in part, for the resistance of BH rats against renal fibrosis. This observation supports the role of oxidative and nitrative mechanisms in DXR toxicity.

Our results obtained in the subgroups of DXR-treated CD and BH rats with similar urinary protein excretion support our view that BH rats are less susceptible to tubulointerstital fibrosis induced by proteinuria. Renal nephrin mRNA expression was similar in the two subgroups, suggesting that the degree of podocyte injury and slit diaphragm leakiness is a primary determinant of proteinuria independent of the genetic background. However, despite similar proteinuria, most markers of renal fibrosis, oxidative stress and inflammation were significantly lower in BH rats. These results support the role of inflammation in proteinuria-induced tubulointerstitial fibrosis.

Resistance mechanisms against DXR nephropathy were studied previously in rat [56] and mouse [57] strains. In spontaneously hypertensive (SHR) rats, cardio- and nephrotoxicity of DXR was more severe than in congenic Wistar-Kyoto (WKY) and in SHR-heart failure rats after subsequent administration of 2 mg/kg DXR on 8 consecutive days. Twelve weeks after the last dose of DXR renal lesions were similar to those in our study with podocyte adhesion leading to glomerulosclerosis and mononuclear infiltration, tubular atrophy and fibrotic matrix expansion in the tubulointerstitium [56]. Severity of these histological changes correlated with strain sensitivity. Similarly to our study, strain differences were partially explained by a difference in the severity of inflammation and arachidonic acid metabolism.

Sensitivity to DXR nephropathy was investigated previously in fibrosis-resistant C57BL/6 and-sensitive BALB/c mice [10]. The difference in susceptibility was attributed to a mutation in the PRKDC gene encoding the **c**atalytic subunit of a DNA activated protein kinase (DNA-PK), a double stranded break repair protein [[10, 58]]. This mutation is also responsible for the severe combined immunodeficiency (SCID) phenotype in mice and rats [59]. DNA-PK expression and activity was also profoundly lower in BALB/c than C57BL/6 mice in a radiation induced apoptosis model [60]. Thus, DNA-PK seems to be crucial in toxic injury models. As inflammation and related oxidative stress also induces DNA damage, the PRKDC gene may play an important role also in our model.

Fibrosis is mediated by myofibroblasts activated by TGF-β1, MCP-1, etc. [61]. In our study, decreased MCP-1 mRNA levels were found in resistant BH rats, which is one of the key chemokines for the migration and infiltration of macrophages to sites of inflammation [62]. The mRNA levels of p91^phox^, also known as NADPH oxidase 2 (NOX2) were also lower in BH rats. NOX2 plays an important role in ROS production of phagocytes and T cells. Furthermore, the mRNA level of p47^phox^, which plays a role in the activation of the NOX2/p22^phox^ complex in the membrane of phagocytes [63], was also milder in BH rats. These findings suggest that less inflammation, accompanied by milder ROS production of the neutrophil cells and macrophages may play a role in the resistance of BH rats against DXR nephropathy.

## Conclusions

In conclusion, resistance of BH rats against renal fibrosis highlighted the role of inflammation-induced oxidative/nitrative stress in chronic podocyte injury leading to glomerulosclerosis and consequent proteinuria in DXR nephropathy. Tubulointerstitial fibrosis is most likely secondary to proteinuria in this model.

## Supporting Information

S1 Data Supplement (PDF)
