# Supplementary material for: Oxidative/Nitrative Stress and Inflammation Drive Progression of Doxorubicin-Induced Renal Fibrosis in Rats as Revealed by Comparing a Normal and a Fibrosis-Resistant Rat Strain
Source: PLoS One. 2015 Jun 18;10(6):e0127090. doi: 10.1371/journal.pone.0127090 (PMC4473269; doi:10.1371/journal.pone.0127090)

Oxidative/nitrative stress and inflammation drive progression of doxorubicin-induced renal fibrosis in rats as revealed by comparing a normal and a fibrosis-resistant rat strain

PONE-D-14-55246 supplementary information

A: Long-term survival		B Short-term survival	
Animal ID	Weeks	Animal ID	Weeks
CD-Surv5	12,286	CD-Surv10	1,571429
CD-Surv5	12	CD-Surv10	1,142857
CD-Surv5	12,571	CD-Surv10	1,571429
CD-Surv5	12,857	CD-Surv10	1,285714
CD-Surv5	12,143	CD-Surv10	1,428571
CD-Surv5	12,143	CD-Surv10	1,428571
CD-Surv5	12,286	CD-Surv10	1,428571
CD-Surv5	10,714	CD-Surv10	1,285714
BH-Surv5	16,429	BH-Surv10	2,428571
BH-Surv5	22,714	BH-Surv10	2,571429
BH-Surv5	12,286	BH-Surv10	1,857143
BH-Surv5	12,429	BH-Surv10	1,857143
BH-Surv5	16	BH-Surv10	1,285714
BH-Surv5	12,571	BH-Surv10	2,571429

A: Body weight changes (g)

Animal ID	Week 0	Week 2	Week 4	Week 6	Week 8
CD/c	347,5	439	503	548	589
CD/c	319	380,5	426	462	491
CD/c	288	361	393	417	437
CD/c	347	427	490	525	548
CD/DXR	376	425	420	414	407
CD/DXR	347,5	397	403	407	385
CD/DXR	307	342	349	366	331
CD/DXR	292,5	345	389	372	306
CD/DXR	282,5	323,5	343	353	317
CD/DXR	304,5	342	375	386	366
CD/DXR	325	376	394	414	333
CD/DXR	349,5	406	413	421	333
BH/c	218	267	299	328	340
BH/c	204	245,5	272	299	314
BH/c	186,5	223	253	270	285
BH/c	211	241	274	294	301
BH/c	202,5	268	280	301	307
BH/DXR	207	228,5	272	285	287
BH/DXR	201	223,5	261	272	272
BH/DXR	213	237,5	260	268	273
BH/DXR	204,5	227	253	270	278
BH/DXR	207,5	228	250	261	267
BH/DXR	191,5	216,5	237	247	250
BH/DXR	205	216	258	273	275
BH/DXR	189,5	207,5	235	244	218
BH/DXR	199	237	242	253	249
BH/DXR	211,5	239	234	246	253

B: Urine protein excretion (mg/24h)

Animal ID	Week 0	Week 2	Week 4	Week 6	Week 8
CD/c	15,15	9,31	35,54	9,48	15,18
CD/c	24,04	21,71	19,18	10,3	21,55
CD/c	4,9	9,33	3,16	8,35	11,69
CD/c	9,02	13,96	5,88	19,02	11,65
CD/DXR	9,48	490,25	582,67	593,25	687,75
CD/DXR	7	285,54	116,38	712,73	657
CD/DXR	13,92	156,56	418,98	325,08	610,71
CD/DXR	10,47	54,02	336,4	465,52	402,86
CD/DXR	12,65	139,67	308,87	457	417,04
CD/DXR	5,67	196,05	329,33	340,37	524,15
CD/DXR	7,52	281,18	312,31	638,47	481,72
CD/DXR	21,11	229,04	318,76	169,94	284,47
BH/c	6,18	13,21	18,69	14,51	14,17
BH/c	3,45	11,89	12,67	16,24	6,94
BH/c	13,25	13,09	16,05	11,05	14,54
BH/c	9,77	12,17	11,41	11	12,97
BH/c	8,33	15,63	10,14	6,46	11,48
BH/DXR	9,73	17,79	147,81	205,68	345,43
BH/DXR	11,58	47,2	188,46	210,97	230,89
BH/DXR	15,63	22,15	304,63	323,33	384,64
BH/DXR	18,5	31,63	27,55	179,29	251,24
BH/DXR	23,22	39,49	157,56	311,8	403,9
BH/DXR	13,41	39,95	123,66	143,67	302,62
BH/DXR	16,75	27,5	41,61	254,16	332,19
BH/DXR	3,6	37,57	193,83	253,6	332,03
BH/DXR	9,49	41,52	234,81	274,13	322
BH/DXR	27,61	24,3	391,21	309,13	346,68

C: Urine NGALn excretion (ug/24h)

Animal ID	Week 0	Week 2	Week 4	Week 6	Week 8
CD/c	2,748	3,363	3,095	2,873	1,285
CD/c	3,638	2,272	2,288	2,706	0,785
CD/c	1,860	2,303	1,585	1,819	0,664
CD/c	1,817	4,415	2,560	3,458	1,163
CD/DXR	1,860	6,753	13,165	19,583	10,274
CD/DXR	1,438	7,958	17,334	20,860	19,276
CD/DXR	1,757	3,671	11,707	18,811	18,965
CD/DXR	1,987	3,707	8,531	10,147	12,081
CD/DXR	2,022	3,767	9,362	11,436	10,131
CD/DXR	4,485	7,113	11,455	11,760	7,400
CD/DXR	1,159	6,590	11,761	13,583	8,072
BH/c	0,932	1,751	4,207	4,925	2,310
BH/c	0,823	2,410	2,396	3,476	0,879
BH/c	1,710	2,361	1,235	2,444	0,816
BH/c	1,513	2,572	1,844	1,042	0,931
BH/c	1,316	2,823	2,333	1,661	0,555
BH/DXR	2,479	3,078	5,534	7,900	3,949
BH/DXR	1,370	2,968	4,224	5,688	5,441
BH/DXR	1,649	1,859	5,752	11,440	6,721
BH/DXR	1,241	2,712	3,531	6,039	5,145
BH/DXR	2,137	6,855	5,587	11,244	6,283
BH/DXR	1,304	5,425	4,072	5,783	5,156
BH/DXR	2,586	3,280	5,096	7,132	4,221
BH/DXR	2,211	2,431	5,417	7,433	3,040
BH/DXR	2,537	4,896	6,700	7,930	10,942
BH/DXR	2,843	2,256	14,021	5,314	5,949

A-C: Glomerular score		D-F: Tubular score		G-I: Inflammation score	
Animal ID	Score value	Animal ID	Score value	Animal ID	Score value
CD/c	0,039	CD/c	0	CD/c	0,1
CD/c	0,071	CD/c	0	CD/c	0,15
CD/c	0,058	CD/c	0	CD/c	0,13
CD/c	0,063	CD/c	0	CD/c	0,3
CD/DXR	0,819	CD/DXR	2,65	CD/DXR	1,81
CD/DXR	1,101	CD/DXR	2,625	CD/DXR	2,1
CD/DXR	0,975	CD/DXR	2,789	CD/DXR	1,87
CD/DXR	0,385	CD/DXR	1	CD/DXR	1,38
CD/DXR	0,619	CD/DXR	1,5	CD/DXR	1,68
CD/DXR	0,905	CD/DXR	1,682	CD/DXR	1,56
CD/DXR	0,831	CD/DXR	2,036	CD/DXR	1,37
CD/DXR	0,679	CD/DXR	1,786	CD/DXR	1,09
BH/c	0,058	BH/c	0	BH/c	0,24
BH/c	0,024	BH/c	0	BH/c	0,17
BH/c	0,032	BH/c	0	BH/c	0,18
BH/c	0,161	BH/c	0	BH/c	0,15
BH/c	0,01	BH/c	0	BH/c	0,17
BH/DXR	0,286	BH/DXR	1,5	BH/DXR	1,14
BH/DXR	0,244	BH/DXR	0,705	BH/DXR	0,87
BH/DXR	0,485	BH/DXR	1,421	BH/DXR	1,43
BH/DXR	0,14	BH/DXR	0,867	BH/DXR	1,14
BH/DXR	0,416	BH/DXR	1,412	BH/DXR	1,28
BH/DXR	0,423	BH/DXR	0,682	BH/DXR	0,85
BH/DXR	0,305	BH/DXR	0,364	BH/DXR	0,88
BH/DXR	0,194	BH/DXR	0,25	BH/DXR	0,84
BH/DXR	0,362	BH/DXR	0,667	BH/DXR	1,02
BH/DXR	0,355	BH/DXR	0,735	BH/DXR	1,11

D: nephrin mRNA levels		F: p47phox mRNA levels	
Animal ID	Rel. Expression	Animal ID	Rel. Expression
CD/c	1,1487	CD/c	0,8939
CD/c	1,3566	CD/c	1,3736
CD/c	0,9075	CD/c	0,7727
CD/c	1,1892	CD/c	0,9598
CD/DXR	0,5946	CD/DXR	2,4014
CD/DXR	0,7371	CD/DXR	1,6898
CD/DXR	0,2449	CD/DXR	2,4291
CD/DXR	0,7423	CD/DXR	2,8717
CD/DXR	0,6417	CD/DXR	4,0102
CD/DXR	0,5285	CD/DXR	4,7314
CD/DXR	0,4830	CD/DXR	5,9876
CD/DXR	0,8467	CD/DXR	5,8697
BH/c	1,0943	BH/c	1,0657
BH/c	0,9727	BH/c	0,8786
BH/c	1,0000	BH/c	0,8990
BH/c	1,0943	BH/c	1,1568
BH/DXR	0,9395	BH/DXR	2,3003
BH/DXR	0,8645	BH/DXR	1,5930
BH/DXR	1,1019	BH/DXR	1,6313
BH/DXR	1,2058	BH/DXR	1,3116
BH/DXR	0,6598	BH/DXR	2,2270
BH/DXR	0,7371	BH/DXR	2,5184
BH/DXR	0,7022	BH/DXR	2,1724
BH/DXR	0,9931	BH/DXR	1,6478

A: TGF-β1 mRNA levels		B: CTGF mRNA levels	
Animal ID	Rel. Expression	Animal ID	Rel. Expression
CD/c	0,9948	CD/c	1,3381
CD/c	0,7471	CD/c	0,9584
CD/c	1,4472	CD/c	1,0499
CD/c	0,8110	CD/c	0,6536
CD/DXR	4,4100	CD/DXR	2,6729
CD/DXR	2,0923	CD/DXR	1,5929
CD/DXR	5,0135	CD/DXR	3,2987
CD/DXR	3,0776	CD/DXR	1,1143
CD/DXR	5,3913	CD/DXR	2,8870
CD/DXR	5,7357	CD/DXR	4,1953
CD/DXR	8,6292	CD/DXR	5,4118
CD/DXR	6,9101	CD/DXR	5,4071
BH/c	0,8762	BH/c	0,5341
BH/c	0,7899	BH/c	0,7790
BH/c	1,1983	BH/c	1,1951
BH/c	1,1356	BH/c	1,4918
BH/DXR	2,7330	BH/DXR	0,6724
BH/DXR	0,9580	BH/DXR	1,0199
BH/DXR	1,1171	BH/DXR	0,3927
BH/DXR	0,9962	BH/DXR	0,5848
BH/DXR	1,0873	BH/DXR	0,5269
BH/DXR	1,0956	BH/DXR	0,5053
BH/DXR	1,9162	BH/DXR	1,3112
BH/DXR	3,5750	BH/DXR	0,5708

L: Fibronectin immunohistochemistry		J: Sirius red staining	
Animal ID	Score value	Animal ID	Score value
CD/c	5,011	CD/c	4,3238
CD/c	4,908	CD/c	1,9925
CD/c	5,782	CD/c	3,7660
CD/c	5,816	CD/c	4,7968
CD/DXR	8,5	CD/DXR	25,6297
CD/DXR	10,028	CD/DXR	33,2227
CD/DXR	6,043	CD/DXR	37,6520
CD/DXR	5,656	CD/DXR	22,5253
CD/DXR	7,187	CD/DXR	16,9033
CD/DXR	10,913	CD/DXR	22,6795
CD/DXR	11,395	CD/DXR	17,2925
CD/DXR	7,38	CD/DXR	16,4887
BH/c	5,088	BH/c	2,1267
BH/c	4,269	BH/c	2,0167
BH/c	4,974	BH/c	2,1543
BH/c	4,767	BH/c	1,5483
BH/c	6,294	BH/c	3,2860
BH/DXR	4,23	BH/DXR	9,6307
BH/DXR	5,067	BH/DXR	14,9730
BH/DXR	4,789	BH/DXR	7,4595
BH/DXR	6,34	BH/DXR	8,1765
BH/DXR	5,168	BH/DXR	11,9640
BH/DXR	6,724	BH/DXR	3,2030
BH/DXR	8,34	BH/DXR	8,6160
BH/DXR	7,11	BH/DXR	16,6300
BH/DXR	6,218	BH/DXR	17,3910
BH/DXR	6,43	BH/DXR	8,7174

**C: COL1A1 mRNA levels**		**E: MCP-1 mRNA levels**		**G: p91phox mRNA levels**	
Animal ID	Rel. Expression	Animal ID	Rel. Expression	Animal ID	Rel. Expression
CD/c	0,6799	CD/c	0,8599	CD/c	0,3962
CD/c	0,4336	CD/c	0,9025	CD/c	0,7658
CD/c	0,7390	CD/c	1,0048	CD/c	1,1540
CD/c	2,1475	CD/c	1,2328	CD/c	1,6841
CD/DXR	13,4632	CD/DXR	10,1546	CD/DXR	2,2691
CD/DXR	11,3633	CD/DXR	3,5263	CD/DXR	5,6451
CD/DXR	15,2286	CD/DXR	6,6650	CD/DXR	10,2238
CD/DXR	16,9505	CD/DXR	4,5189	CD/DXR	7,0318
CD/DXR	19,5712	CD/DXR	10,5057	CD/DXR	11,3442
CD/DXR	26,6740	CD/DXR	9,4225	CD/DXR	12,1854
CD/DXR	30,7756	CD/DXR	12,1021	CD/DXR	12,0952
CD/DXR	25,0576	CD/DXR	9,7915	CD/DXR	12,2678
BH/c	1,1609	BH/c	0,5757	BH/c	1,3664
BH/c	0,8085	BH/c	0,9859	BH/c	0,8964
BH/c	1,1795	BH/c	0,9527	BH/c	1,1007
BH/c	0,8510	BH/c	1,4857	BH/c	0,6365
BH/DXR	3,7871	BH/DXR	2,5688	BH/DXR	1,3275
BH/DXR	2,4950	BH/DXR	2,2583	BH/DXR	1,9489
BH/DXR	4,0362	BH/DXR	4,8737	BH/DXR	1,8694
BH/DXR	1,9650	BH/DXR	2,3242	BH/DXR	2,9488
BH/DXR	8,9460	BH/DXR	3,2348	BH/DXR	1,5000
BH/DXR	2,1653	BH/DXR	3,2400	BH/DXR	2,1397
BH/DXR	4,8373	BH/DXR	3,8614	BH/DXR	3,5987
BH/DXR	6,3814	BH/DXR	3,1556	BH/DXR	3,0479

Fibronectin immunohistochemistry

BH/c:

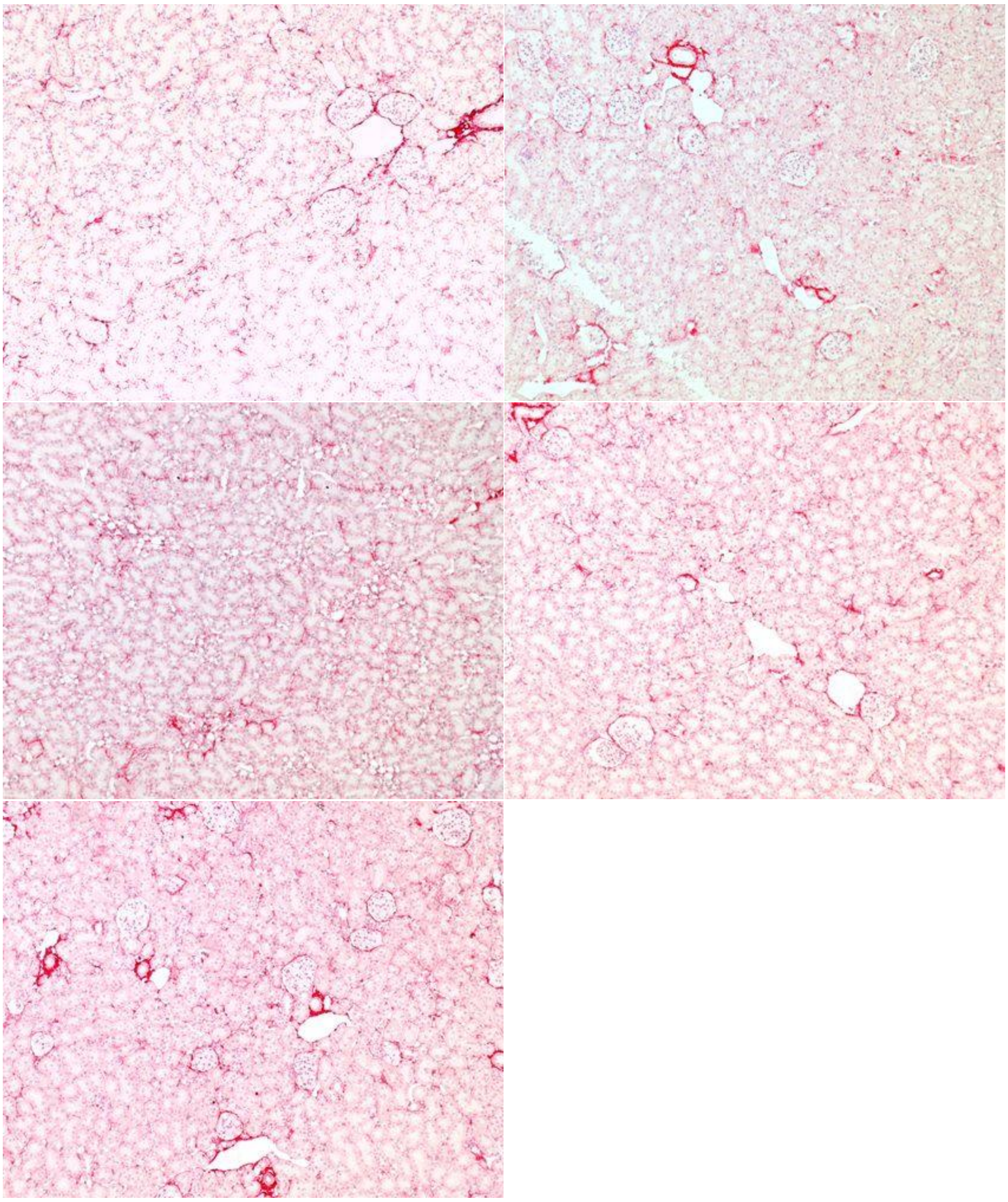

CD/c:

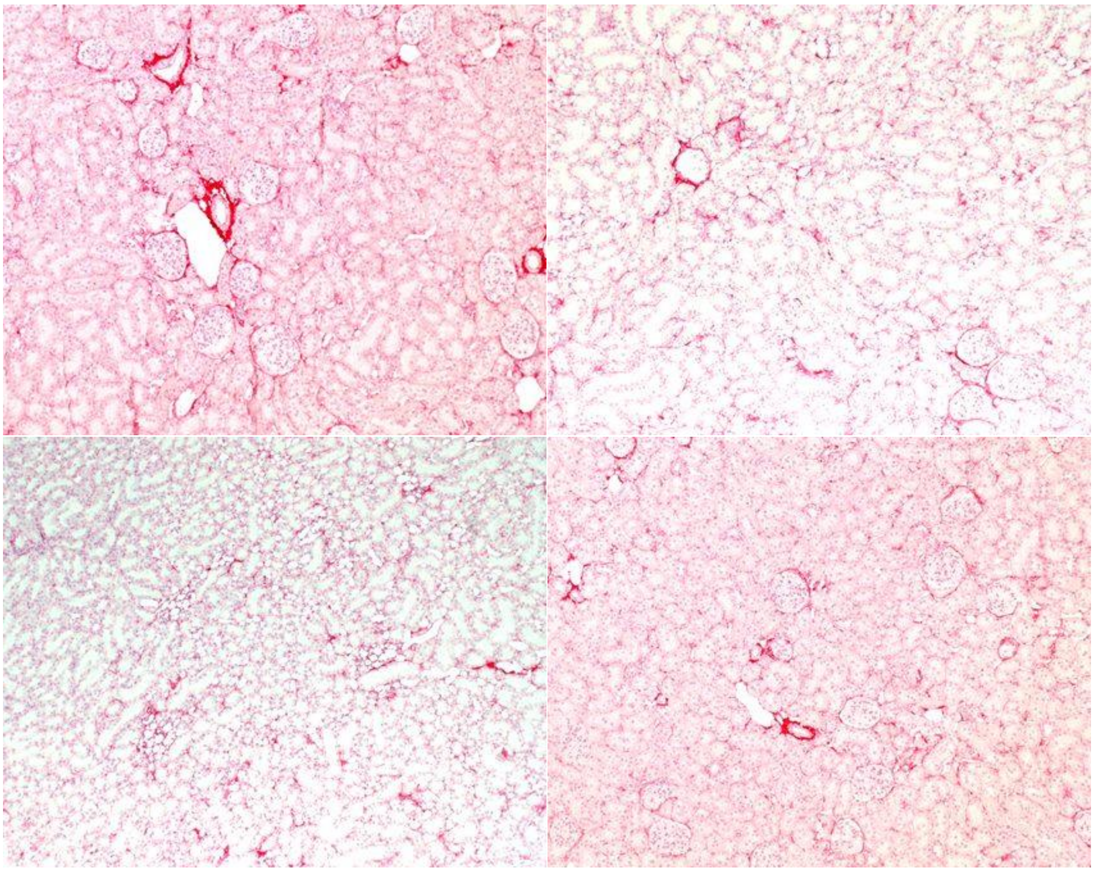

BH/DXR:

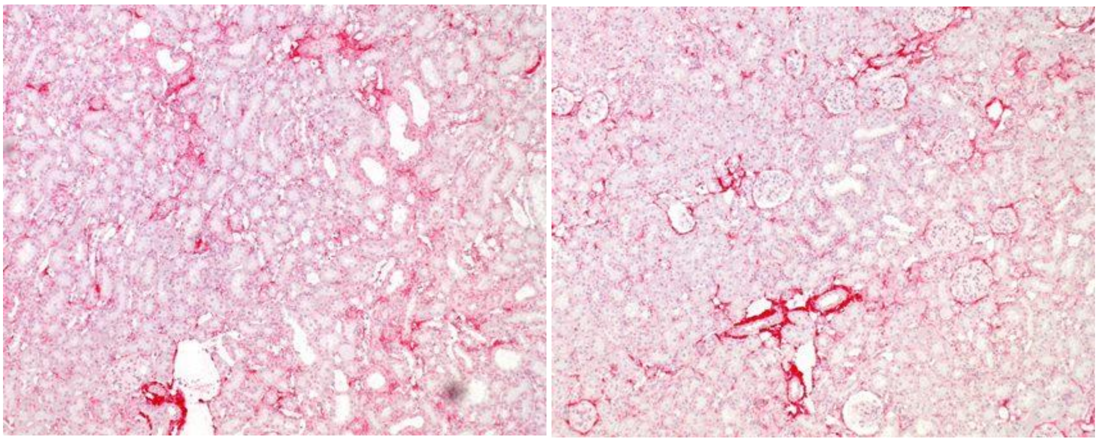

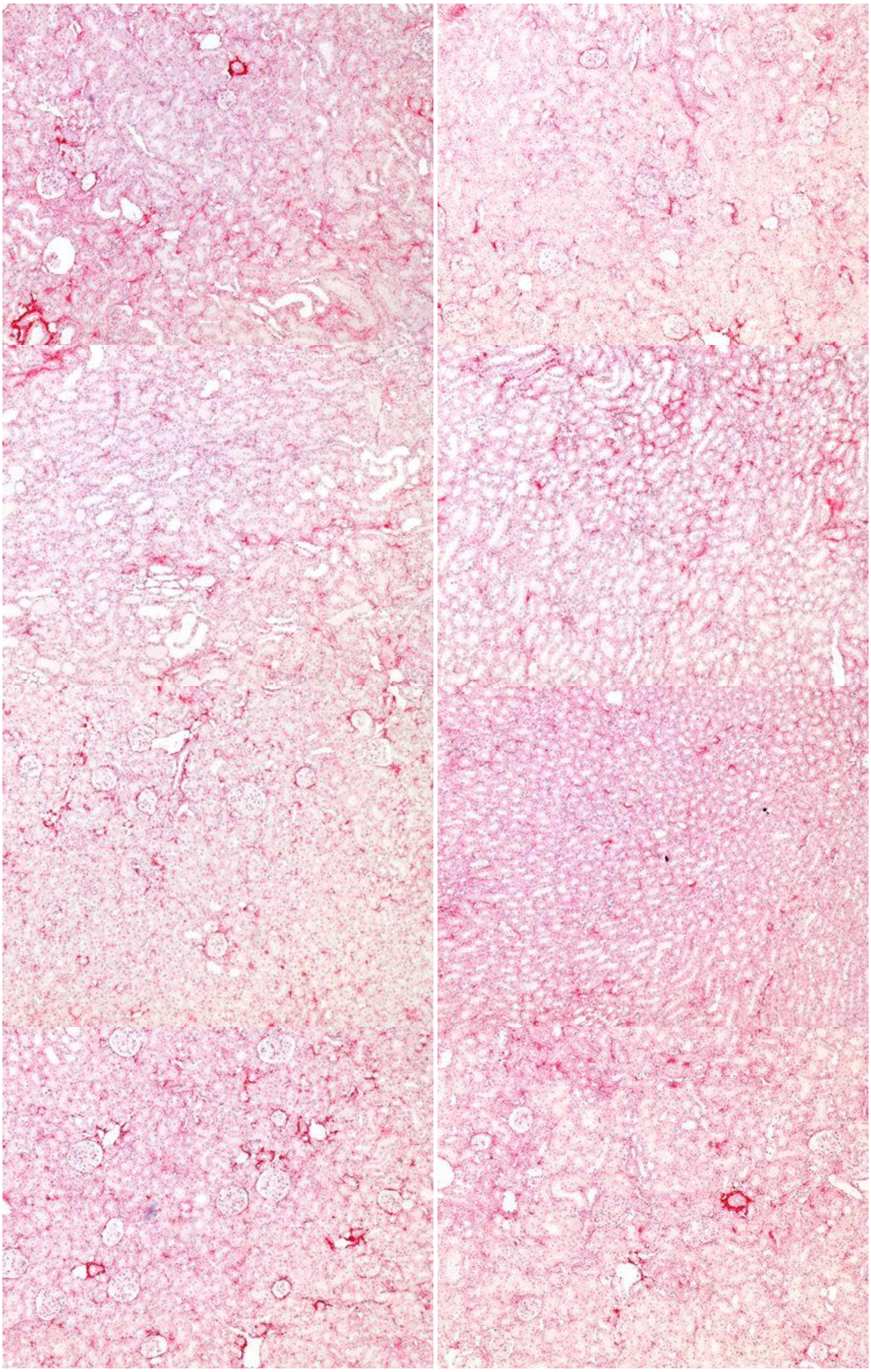

CD/DXR:

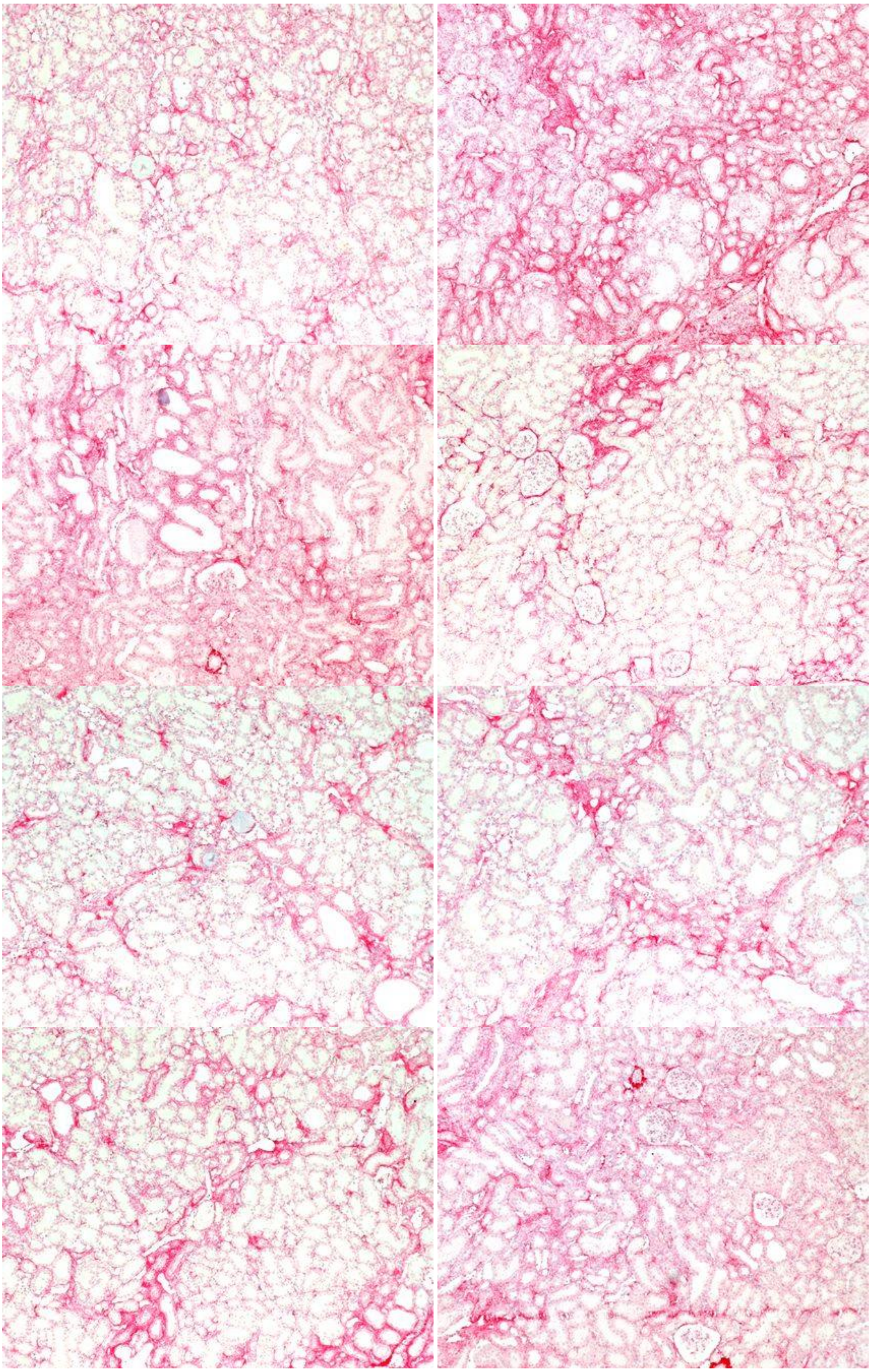

Sirius red staining

BH/c:

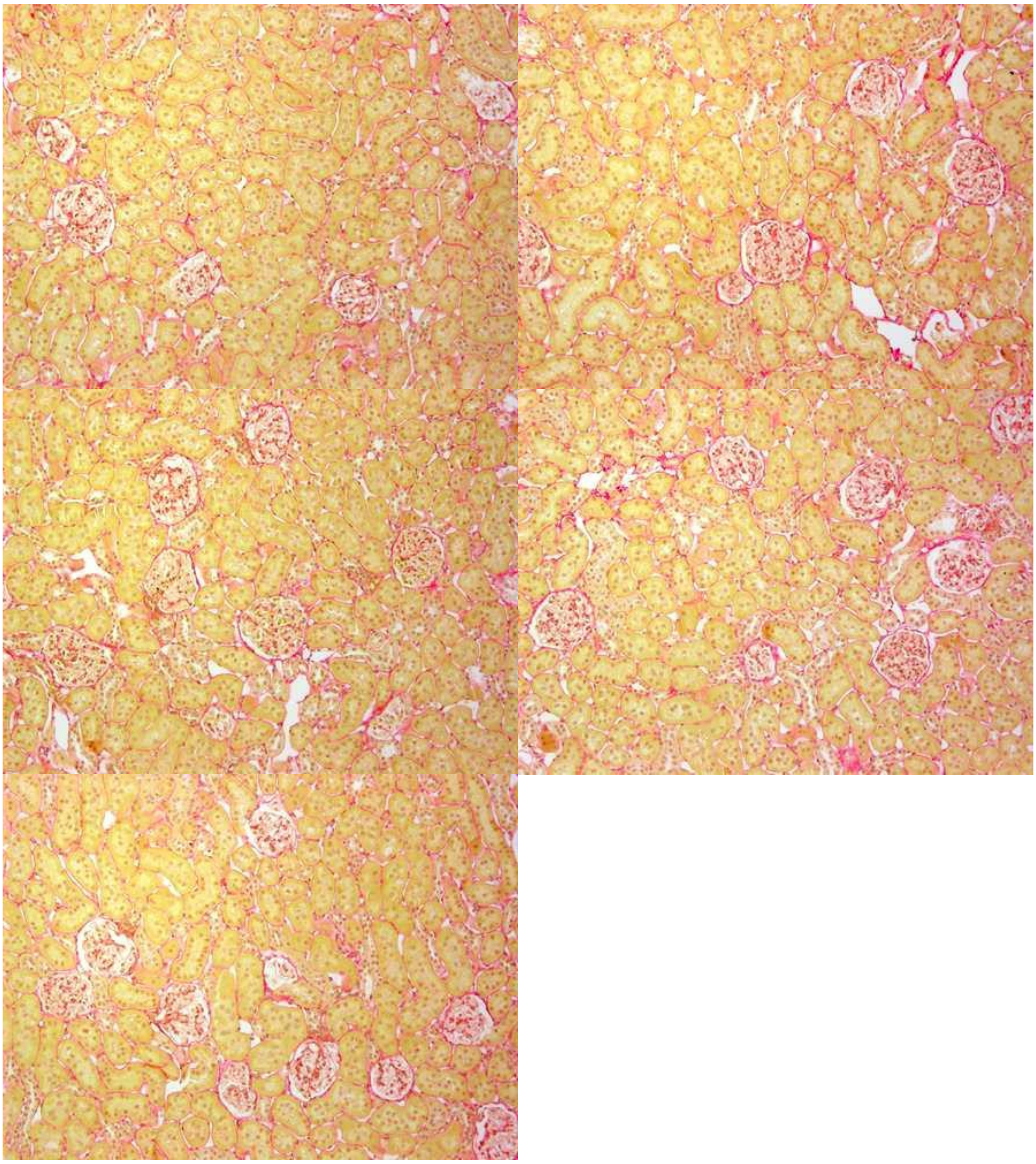

CD/c:

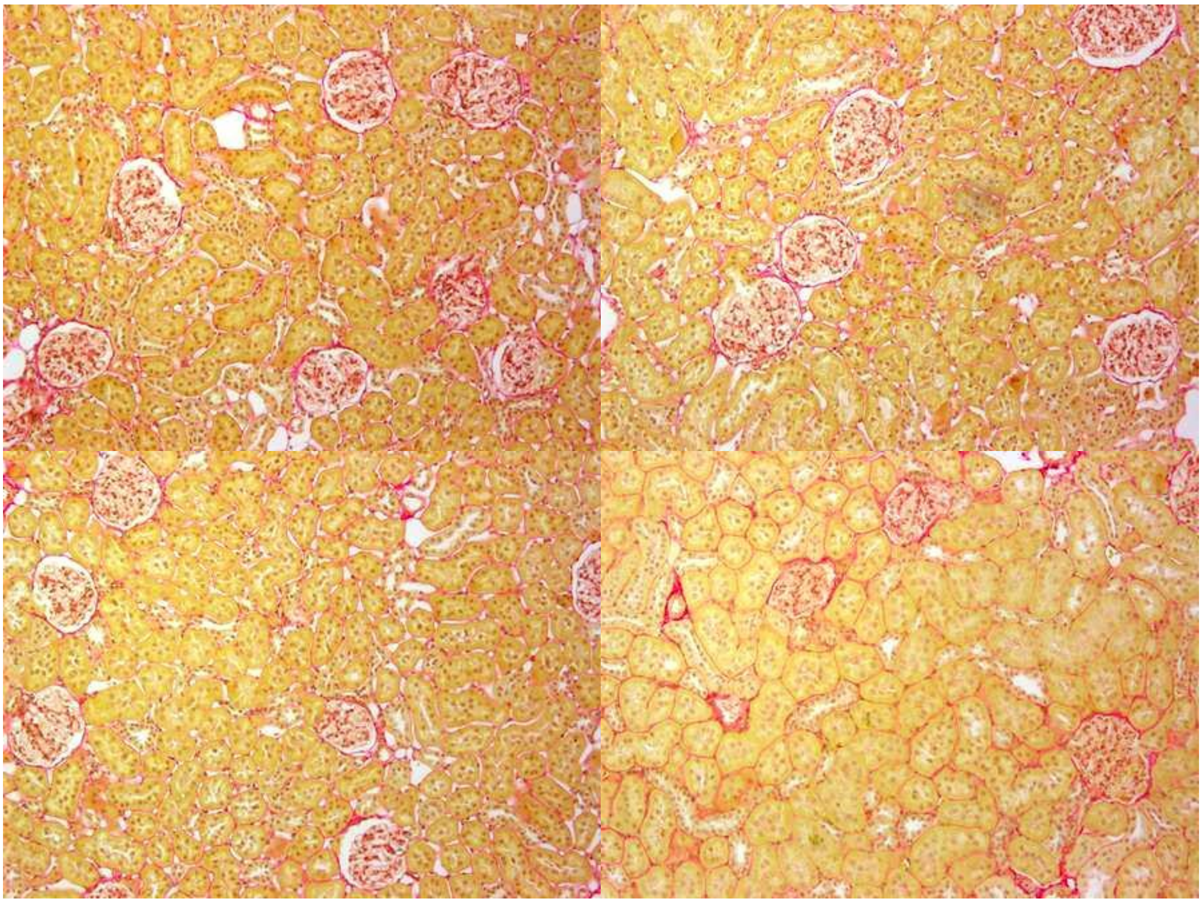

BH/DXR:

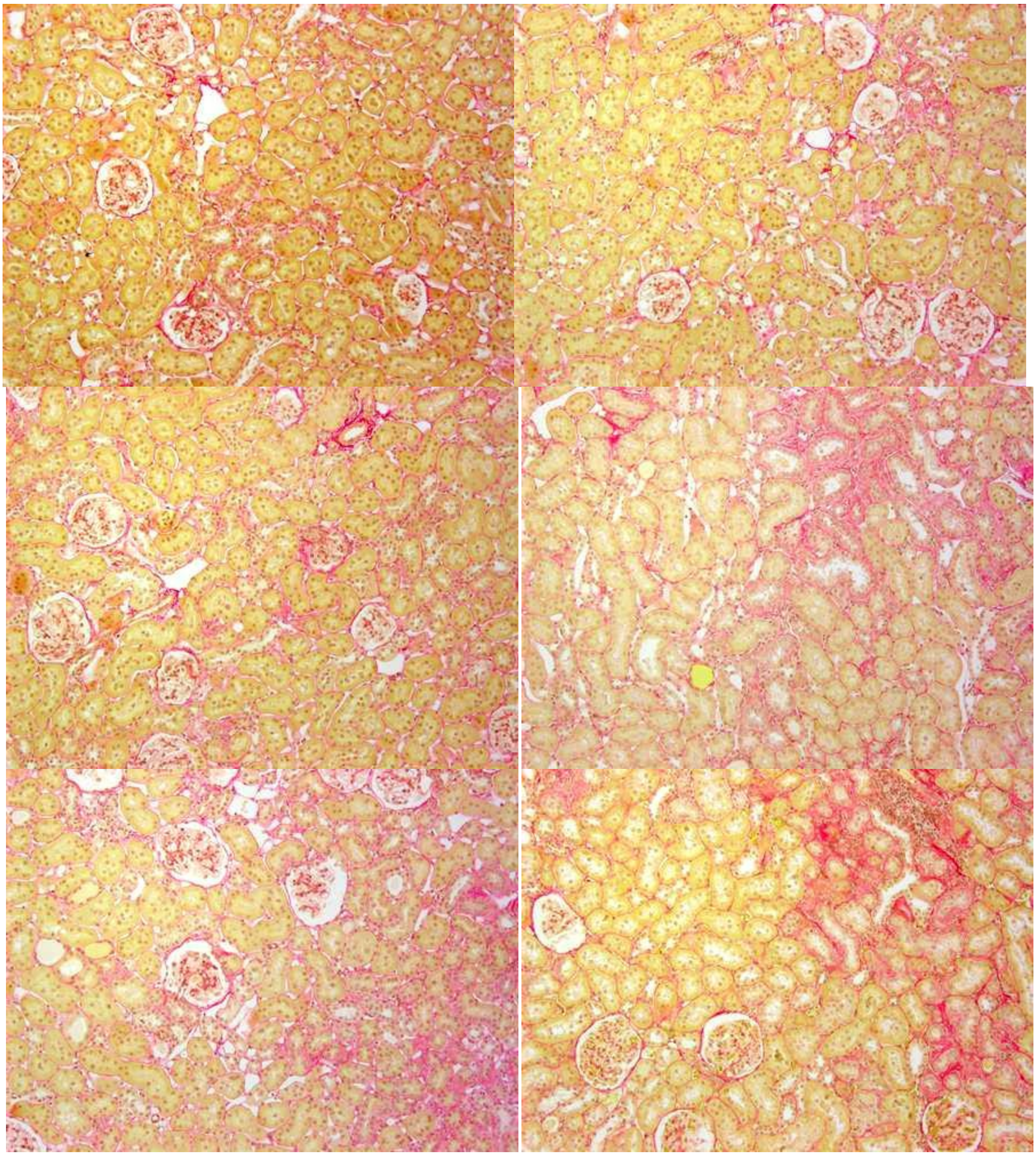

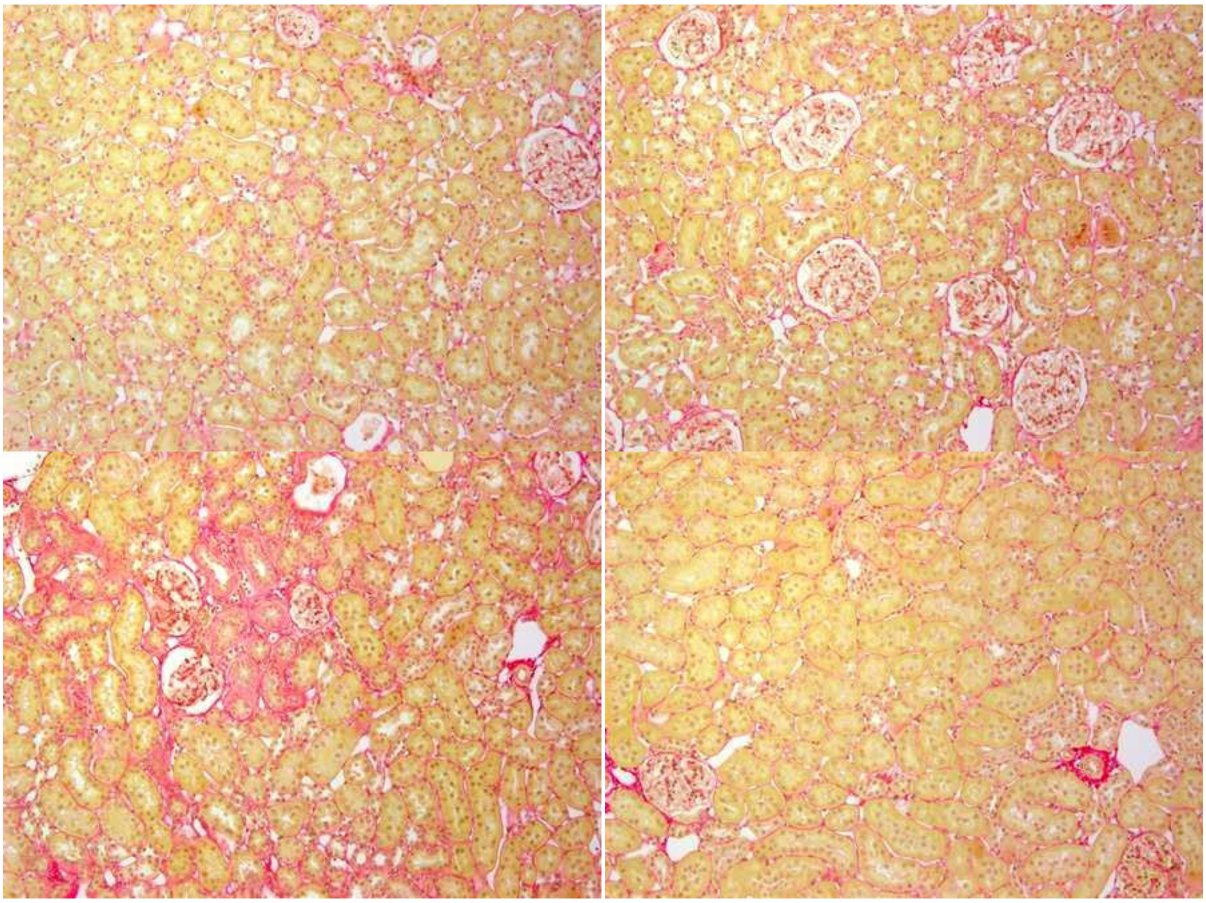

CD/DXR:

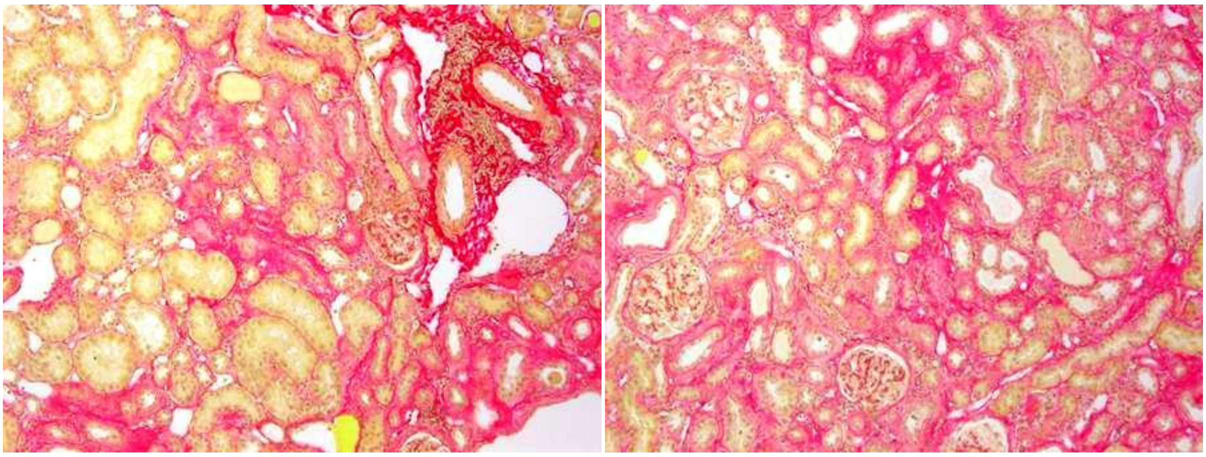

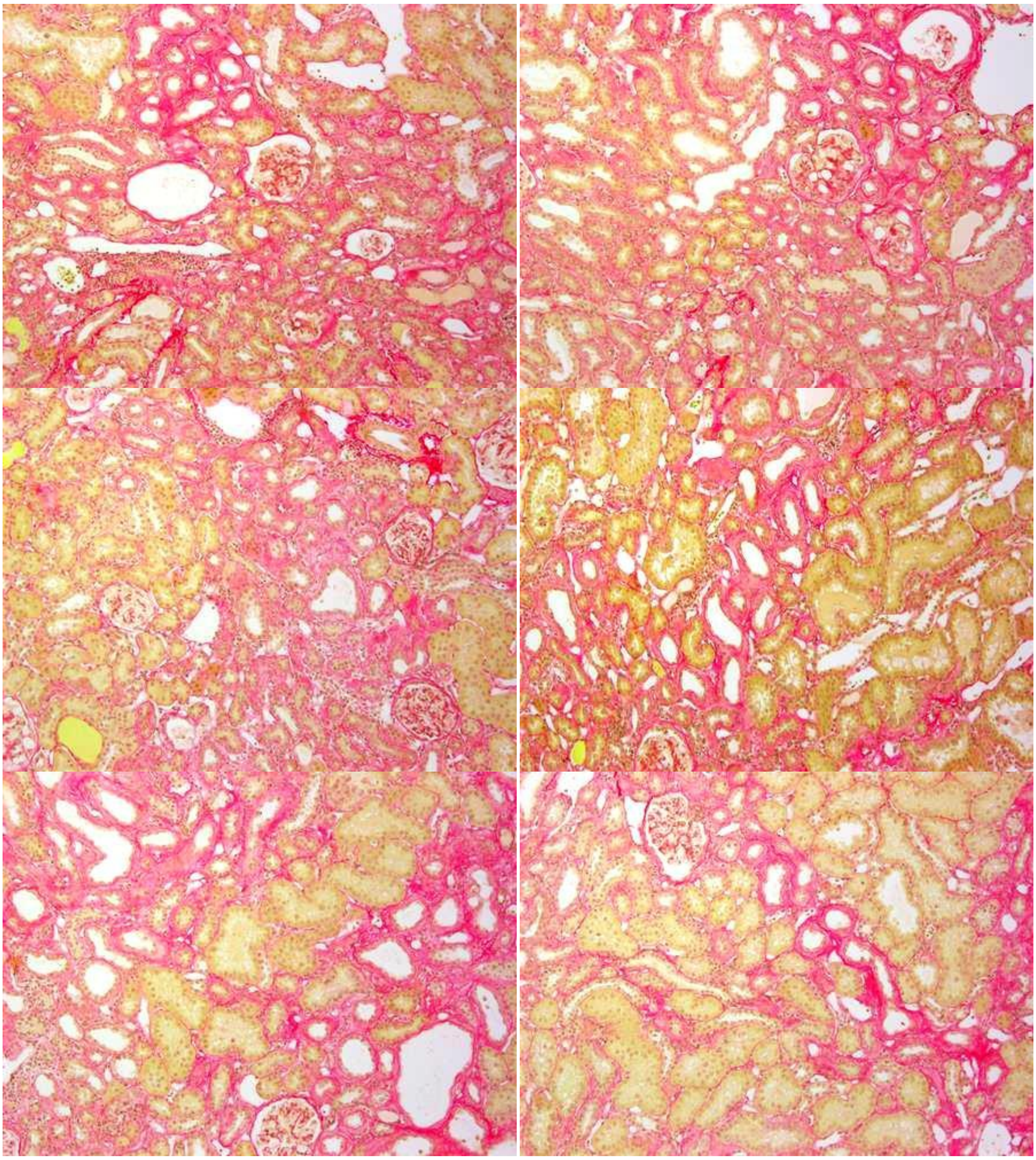

Periodic Acid Schiff (PAS) staining

BH/c:

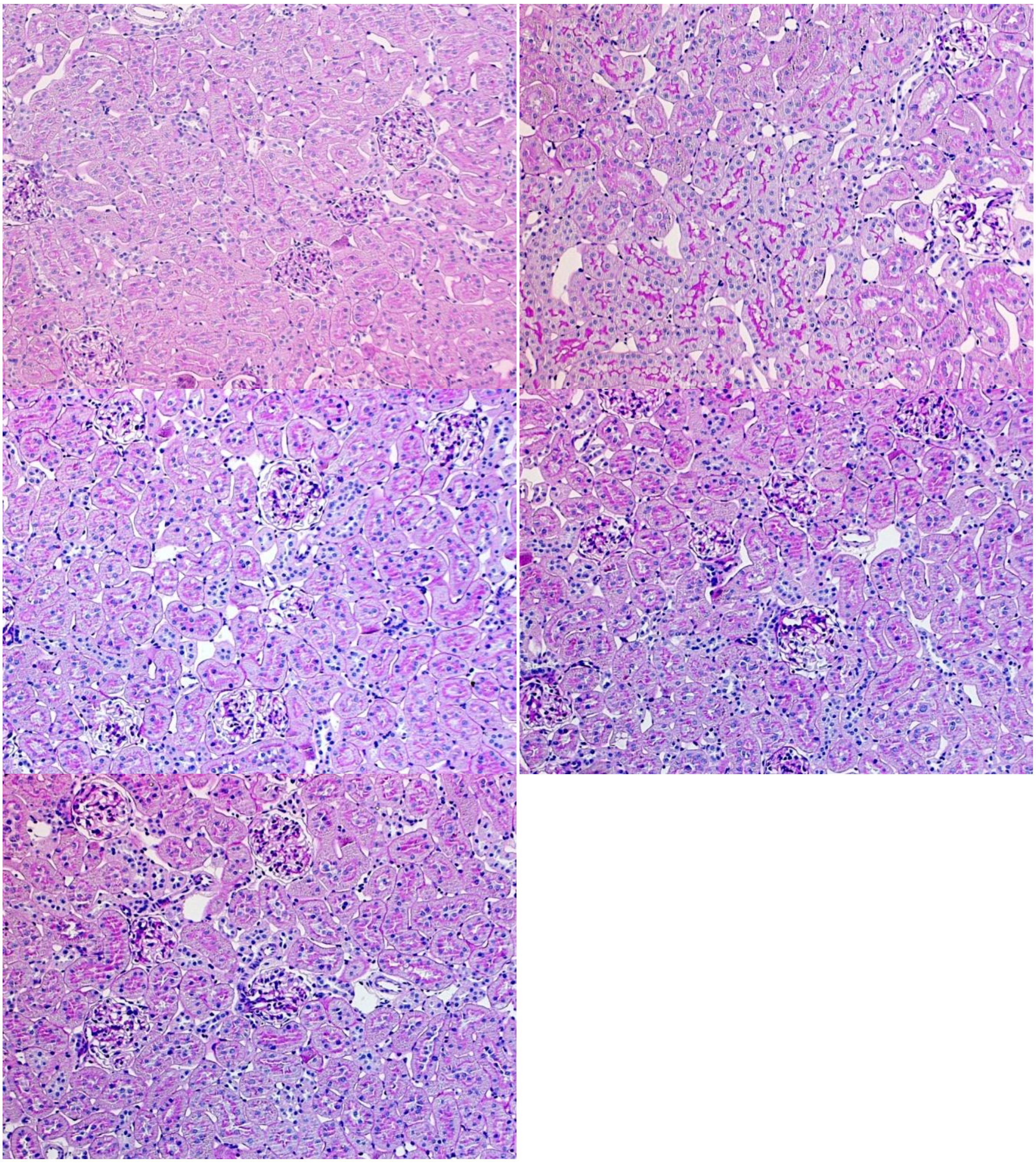

CD/c:

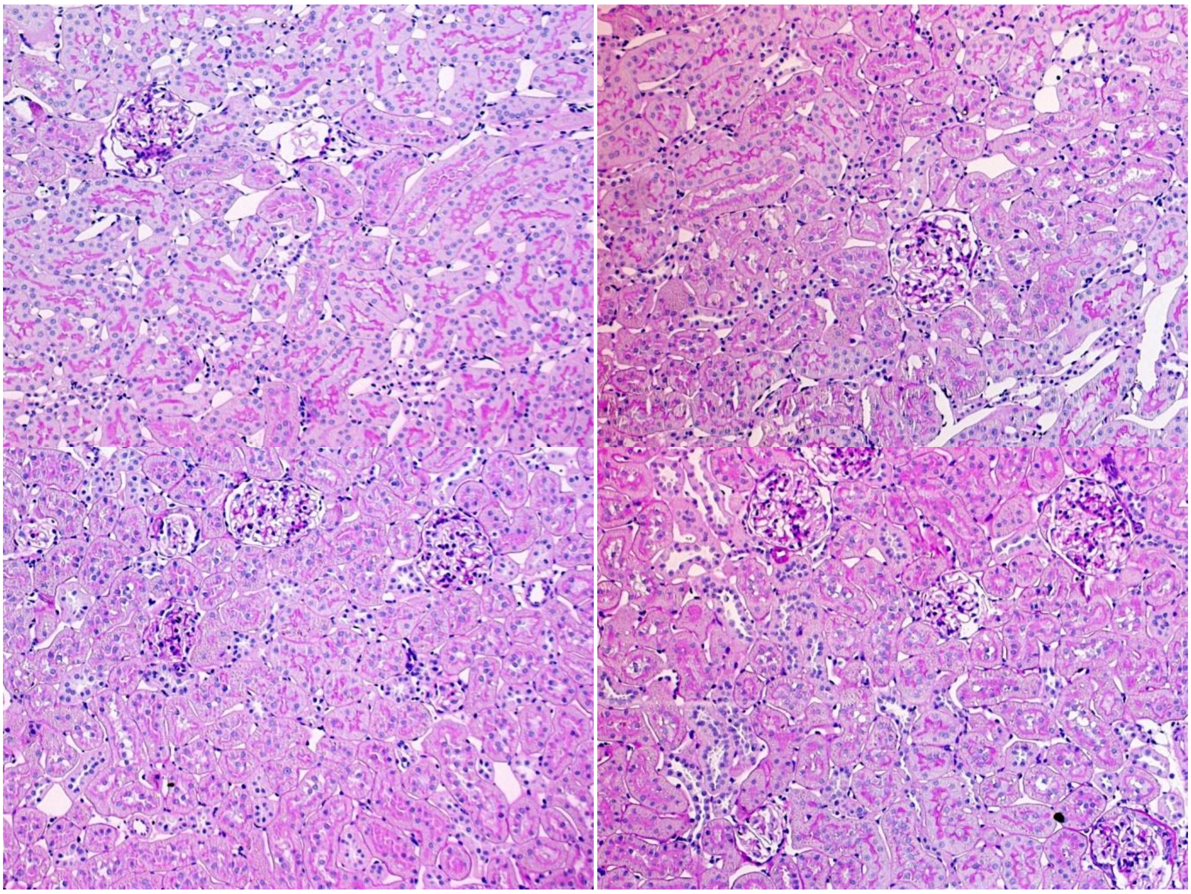

BH/DXR:

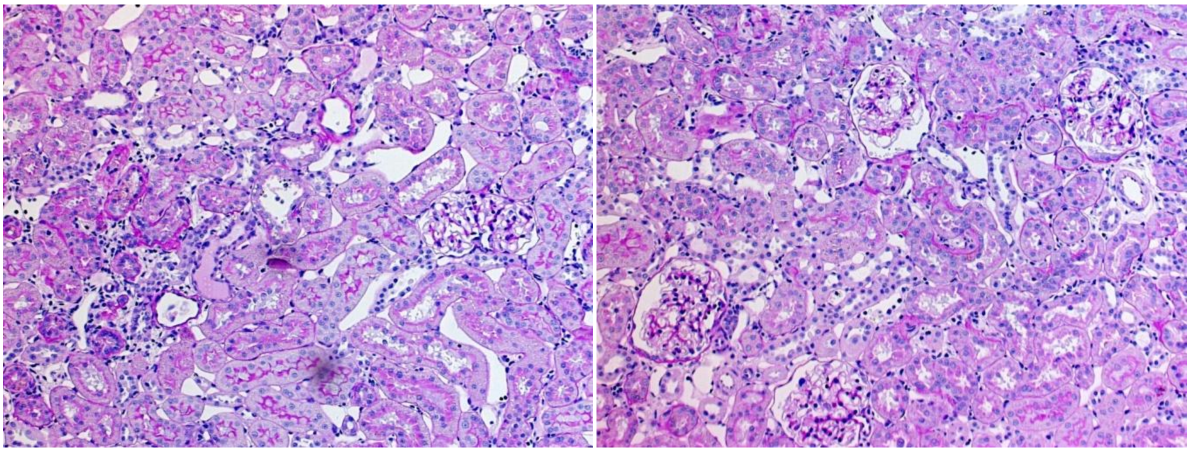

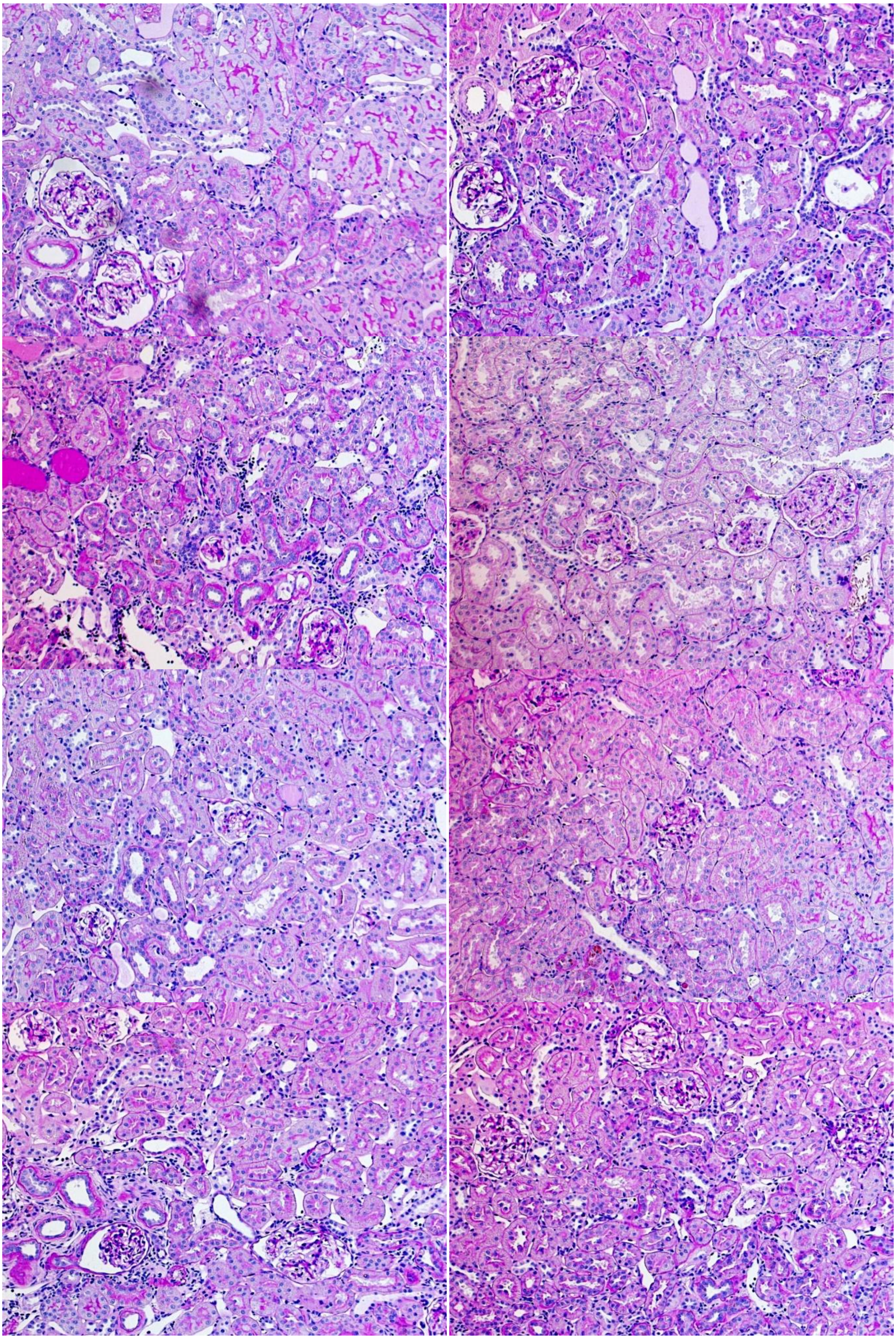

CD/DXR:

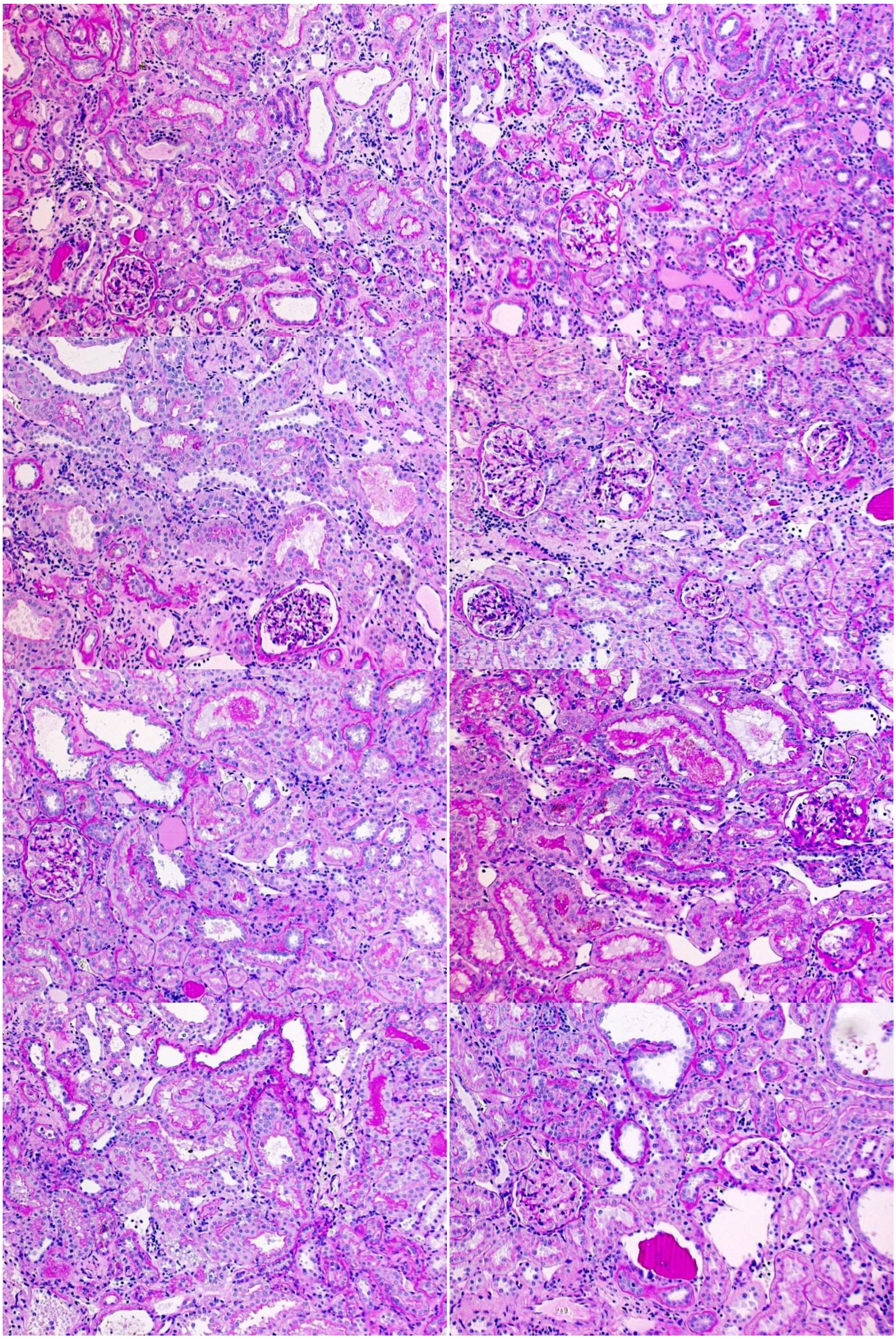

Hematoxylin eosin staining

BH/c:

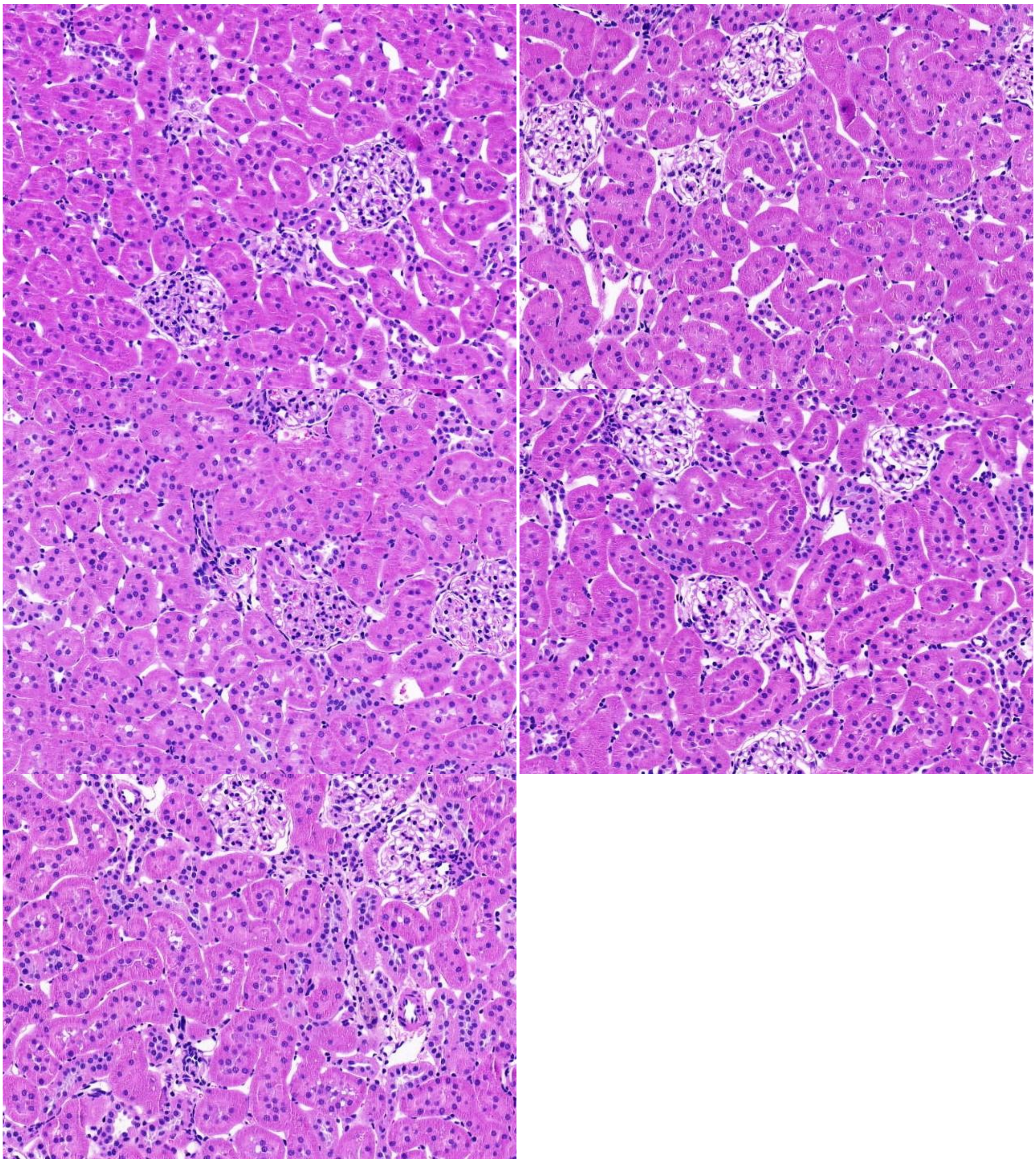

CD/c:

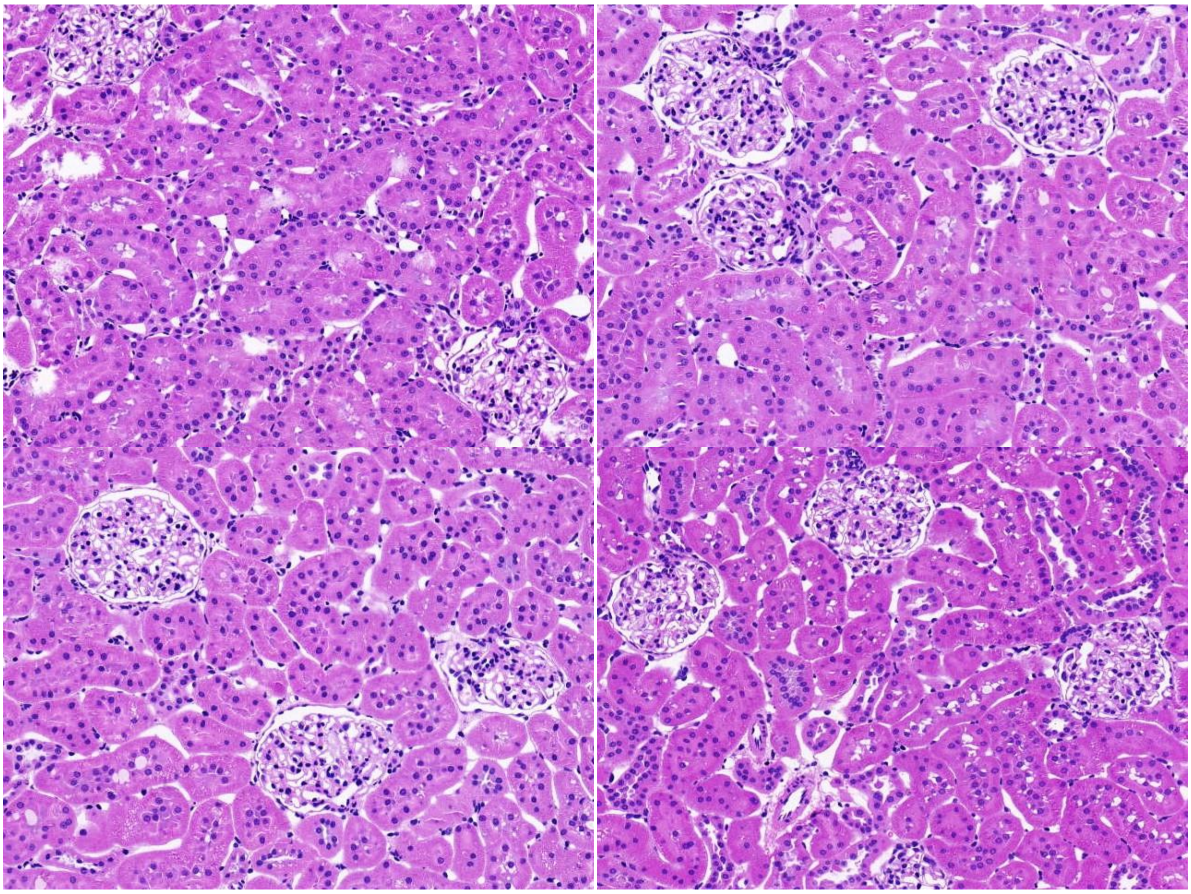

BH/DXR:

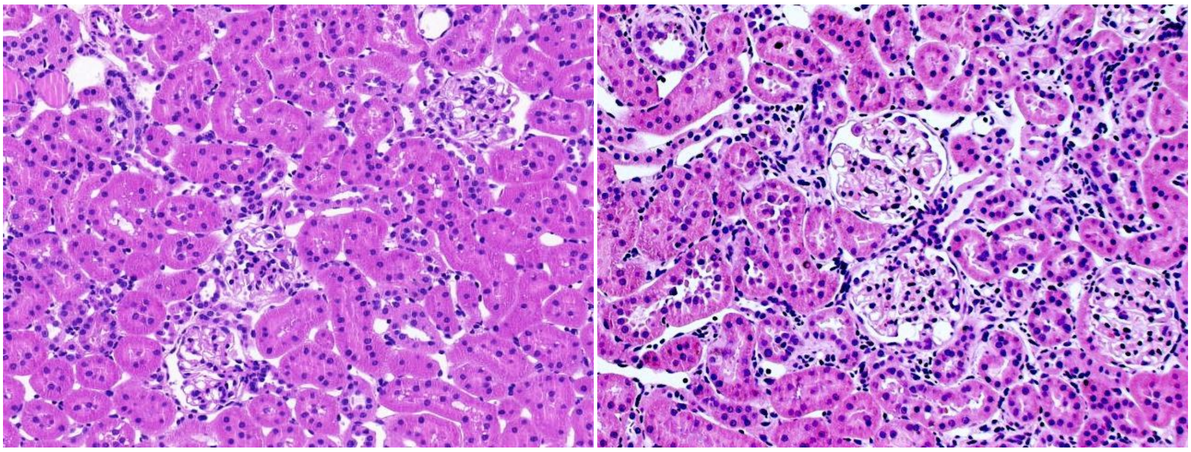

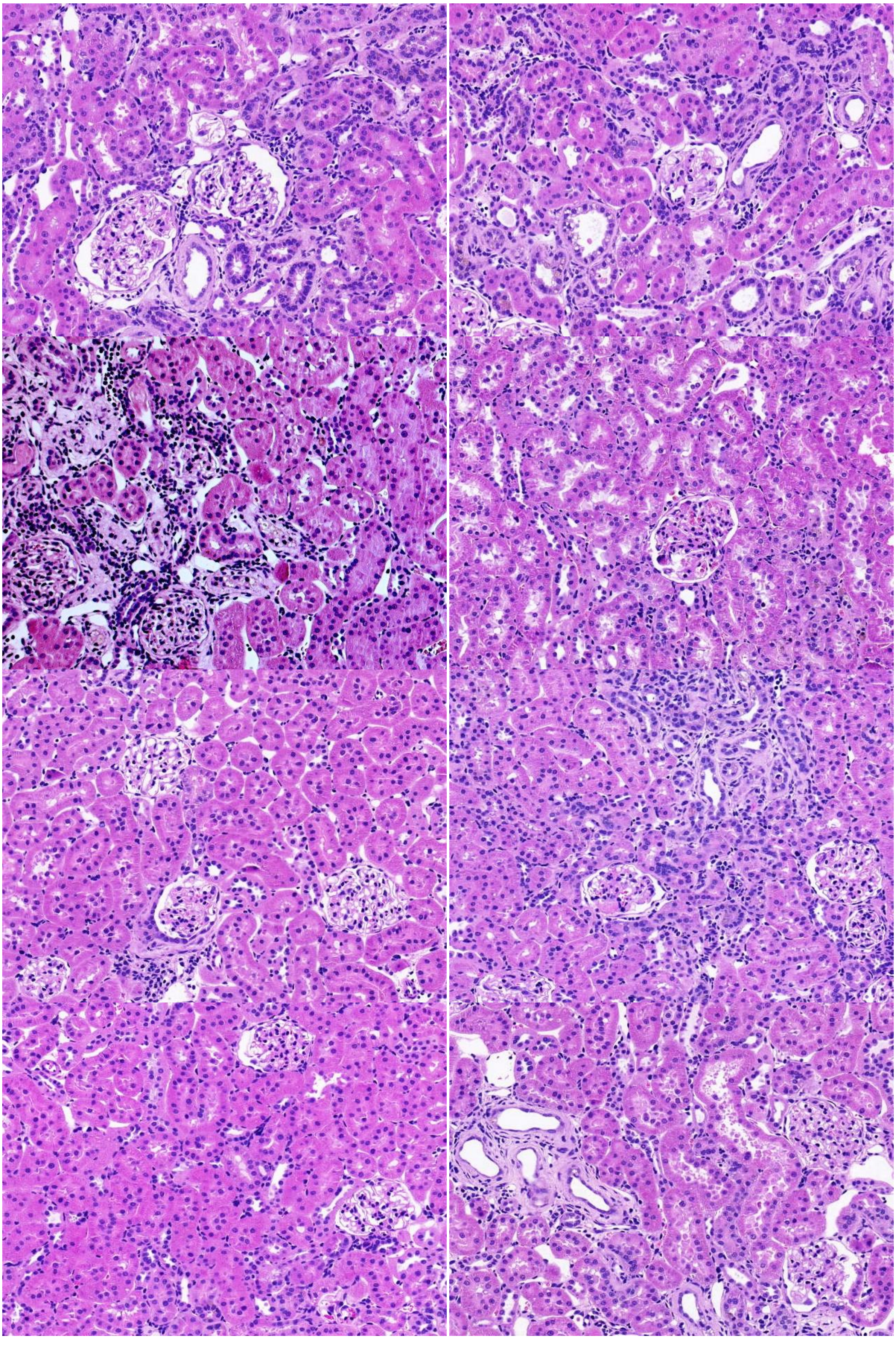

CD/DXR:

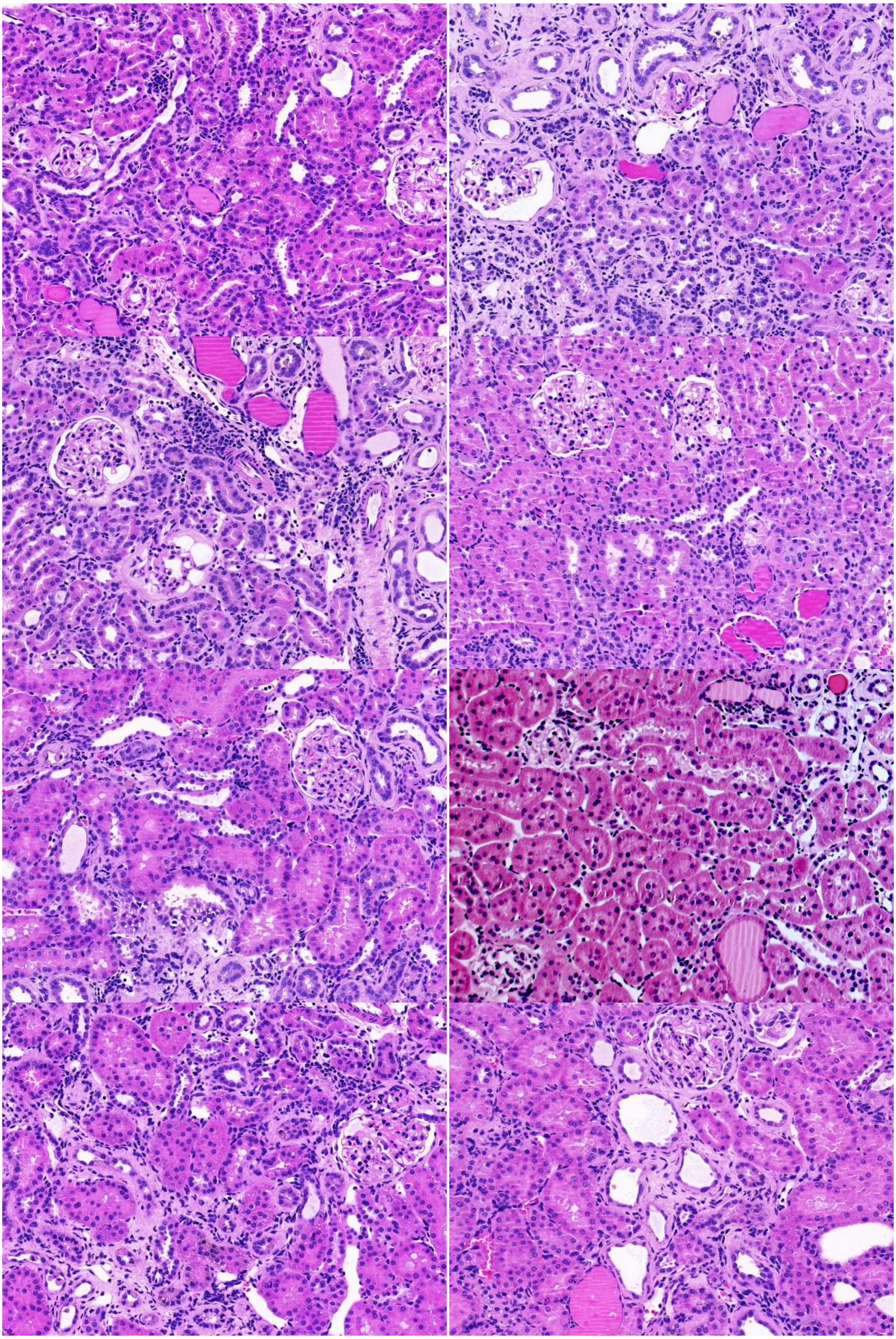

Supplement: S1 Data Supplement — (PDF) [file pone.0127090.s001.pdf]
